# Dual Integrating Oxygen and Sulphur on Surface of CoTe Nanorods Triggers Enhanced Oxygen Evolution Reaction

**DOI:** 10.1002/advs.202206204

**Published:** 2023-01-26

**Authors:** Xin Wang, Zhelin Mao, Xin Mao, Ximiao Hu, Feiyue Gao, Minrui Gao, Qi‐Long Wu, Xiao Lyu, Aijun Du, Xiangsheng Xu, Yi Jia, Lei Wang

**Affiliations:** ^1^ College of Chemical Engineering Zhejiang University of Technology Hangzhou 310032 P. R. China; ^2^ School of Chemistry, Physics and Mechanical Engineering Queensland University of Technology Brisbane QLD 4000 Australia; ^3^ Department of Chemistry Institute of Biomimetic Materials & Chemistry Anhui Engineering Laboratory of Biomimetic Materials Division of Nanomaterials & Chemistry Hefei National Research Center for Physical Sciences at the Microscale Institute of Energy Hefei Comprehensive National Science Center University of Science and Technology of China Hefei 230026 P. R. China; ^4^ Intelligent Polymer Research Institute and ARC Centre of Excellence for Electromaterials Science Australian Institute for Innovative Materials University of Wollongong Wollongong NSW 2500 Australia; ^5^ School of Materials Science and Engineering Shenyang Ligong University Shenyang 110159 P. R. China

**Keywords:** doping, electrocatalysis, oxygen evolution reaction, surface oxidization, transition metal telluride

## Abstract

The bottleneck of large‐scale implementation of electrocatalytic water‐splitting technology lies in lacking inexpensive, efficient, and durable catalysts to accelerate the sluggish oxygen evolution reaction kinetics. Owing to more metallic features, transition metal telluride (TMT) with good electronic conductivity holds promising potential as an ideal type of electrocatalysts for oxygen evolution reaction (OER), whereas most TMTs reported up to now still show unsatisfactory OER performance that is far below corresponding sulfide and selenide counterparts. Here, the activation and stabilization of cobalt telluride (CoTe) nanoarrays toward OER through dual integration of sulfur (S) doping and surface oxidization is reported. The as‐synthesized CoO@S‐CoTe catalyst exhibits a low overpotential of only 246 mV at 10 mA cm^−2^ and a long‐term stability of more than 36 h, outperforming commercial RuO_2_ and other reported telluride‐based OER catalysts. The combined experimental and theoretical results reveal that the enhanced OER performance stems from increased active sites exposure, improved charge transfer ability, and optimized electronic state. This work will provide a valuable guidance to release the catalytic potential of telluride‐based OER catalysts via interface modulating engineering.

## Graphical abstract

Surface oxidized S‐doped CoTe nanoarrays are designed and synthesized on nickel foam. The obtained best CoO@S‐CoTe catalyst even exhibits an excellent OER performance, in terms of over potential and stability, that can be comparable to benchmark RuO_2_. The synergistic doping and surface oxidation strategies are provided to solve the activity and structural stability problems of OER telluride catalysts.

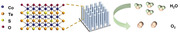

## 1 Introduction

Electrocatalytic water splitting, consisted of oxygen evolution reaction (OER) and hydrogen evolution reaction (HER), is a promising technology to produce clean hydrogen to tackle the growing energy shortage and environmental problems.^[^ ^1^ ^]^ At present, the large‐scale deployment of water‐splitting device is still severely hindered by the sluggish anodic OER kinetics.^[^ ^2^ ^]^ Although noble‐metal oxides, like RuO_2_ and IrO_2_, have been widely used as workhorse catalysts to accelerate the process, the high cost, rare reserves, and poor stability limit their practical utilization.^[^ ^3^ ^]^ Therefore, there is an urgent need to develop cost‐efficient alternatives with high activity and durability for realizing affordable water electrolysis industry.

Transition metal telluride (TMT), as an important member of transition metal chalcogenides (TMCs), has stimulated intense attention in recent years due to its structural diversity and unique electronic state.^[^ ^4^ ^]^ As the heaviest, nonradioactive member of chalcogens, Te possesses more metallic character than the lighter elements in the same family, including O, S, and Se.^[^ ^5^ ^]^ This ensures high electron conductivity and easy electron transfer in electrochemical process.^[^ ^6^ ^]^ The OER activity of chalcogenides was previously demonstrated to have a positive correlation with the degree of covalency in metal–chalcogen bond.^[^ ^7^ ^]^ Accordingly, TMT materials should be ideal OER catalysts that are superior to other nonradioactive TMCs. However, the reality is disappointing and most reported TMTs until now still exhibit unsatisfactory OER performance that is far below corresponding sulfide and selenide counterparts. Therefore, it is urgent but still challenging to exploit more efficient structural modulation strategy to unlock the catalytic power of TMT materials.

Fundamentally, the OER activity is closely related with the adsorption behavior of intermediates during OER process, which is highly relied on the electronic structure of catalyst materials.^[^ ^8^ ^]^ Based on early studies and our density functional theory (DFT) calculation, pure transition metal telluride, like cobalt telluride, needs to overcome the large energy barrier for transforming O* into key OOH* intermediate when driving OER, thus restricting its practical catalytic performance.^[^ ^9^ ^]^ Doping a secondary nonmetal element has been proved to be effective in modulating electronic configuration of OER electrocatalysts, but this method cannot prevent self‐reconstruction for chalcogenides under oxidation potential, leading to shortened catalysts’ lifetime and the ambiguity in identifying the real catalytic mechanism.^[^ ^10^ ^]^ Recently, rational surface oxidation in metal chalcogenides was employed to successfully address this challenge. For example, Domen and co‐workers reported that oxysulfides were more stable against self‐oxidation than corresponding sulfides due to the hydrated O‐2p and S‐3p orbitals.^[^ ^11^ ^]^ Our previous work confirmed that pre‐constructing surface oxide layer in transition metal selenides could significantly improve the OER activity and durability.^[^ ^12^ ^]^ Inspired by above analysis, it is believed that combination of nonmetal element doping and surface oxidation may provide a promising way to strikingly enhance the OER performance for telluride catalysts. This can explain why we design the unique CoTe‐based OER catalysts with dual incorporation of oxygen and sulfur. Moreover, the well‐defined interface as active sites can serve more easily as the model system for atomic‐level insight into the catalytic reaction mechanism in telluride system.

Herein, we design and synthesize partially oxidized S‐doped CoTe nanoarrays on Ni foam (noted as CoO@S‐CoTe) as a robust and efficient electrocatalyst for OER in alkaline electrolyte. At the atomic level, S atoms doping facilitates the transfer of charge from the CoO to the internal Co site. As a result, the obtained CoO@S‐CoTe catalyst exhibited superior performance, reaching the current density of 10 mA cm^−2^ by applying substantially low overpotential of only 246 mV, which surpasses commercial RuO_2_ and other reported telluride‐based OER catalysts. Moreover, no obvious reconstruction phenomenon can be observed after 36 h of long‐term stability measurement, demonstrating the structural robustness of such CoTe‐based catalyst. Our work provide a new synergistic doping and surface oxidation strategy to solve the activity and structural stability problem of telluride OER catalysts.

## 2 Results and Discussion

**Figure 1:**
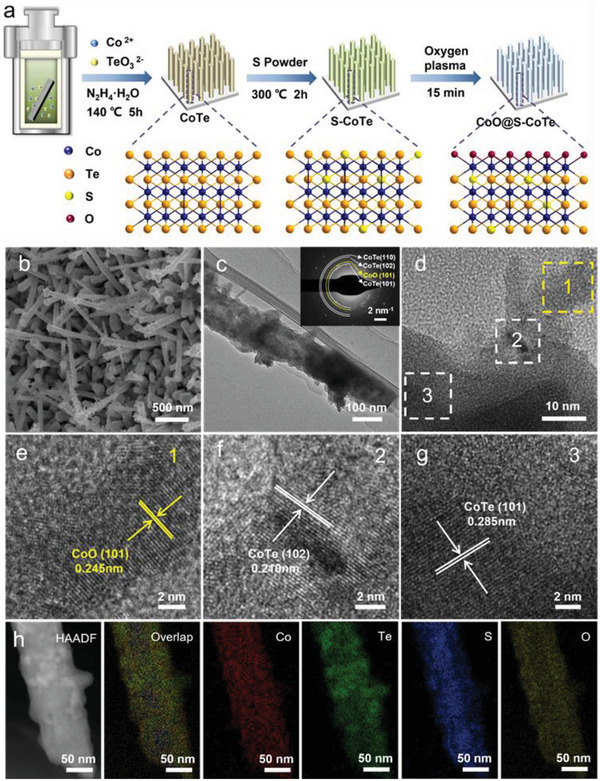
a) Schematic diagram of the synthesis of CoO@S‐CoTe. b) SEM, c) TEM image and the corresponding SAED pattern (inset), d–g) HRTEM images, h) HAADF‐STEM and EDX element mapping images of CoO@S‐CoTe.

**Figure** **1**a shows the synthetic strategies of CoO@S‐CoTe. First, CoTe nanoarrays are grown on the nickel foam (NF) through a hydrothermal method, in which N_2_H_4_ served as a reducing agent to react with Na_2_TeO_3_ and CoSO_4_ towards the formation of CoTe.^[^ ^9^ ^]^ S atoms were then incorporated into CoO@S‐CoTe via Chemical Vapor Deposition (CVD) method which is very suitable for in situ doping.^[^ ^13^ ^]^ After that, the oxide layer is constructed on the CoO@S‐CoTe surface through O plasma treatment. A combination of scanning electron microscopy (SEM), and energy‐dispersive X‐ray spectroscopy (EDS) mappings (Figures S1 and S2, Supporting Information) reveal that CoTe nanorods have been grown on the NF surface. Subsequently, the heteroatom doping was implemented at 300 °C under Ar atmosphere. The morphology of S‐CoTe did not change obviously after sulfur incorporation into CoTe (Figure S3, Supporting Information). The transmission electron microscopy (TEM) images and selected area electron diffraction (SAED) further confirmed the successful preparation of nanorod‐morphology CoTe and S‐CoTe(Figures S4–S6, Supporting Information). Finally, the S‐CoTe were subsequently treated with O plasma, and the resulting material (CoO@S‐CoTe) showed a rod‐like shape with a diameter of ≈100 nm (Figure 1b). TEM image in Figure 1c exhibits that CoO@S‐CoTe has a rough surface which is beneficial to the exposure of active sites and electron transfer. Many different lattice fringes are observed in high‐resolution TEM (HRTEM) images (Figure 1d–g), indicating the polycrystalline character of CoO@S‐CoTe. The lattice fringes with a spacing of 0.210 nm and 0.285 nm correspond to the (102) and (101) planes of CoTe while the lattice spacing of 0.245 nm is assigned to the (101) plane of CoO. SAED rings in Figure 1c shows the (101), (102), (110) planes of CoTe and CoO (101) planes, which are consistent with HRTEM results. EDS mapping images in Figure 1h illuminate the homogeneous distribution of Co, Te, S, and O throughout the nanorod. After O plasma treatment, the signal of S 2p was weakened compared to the previous one, indicating that the S and Te atoms on the surface were replaced by oxygen atoms (Figure S7, Supporting Information).

**Figure 2:**
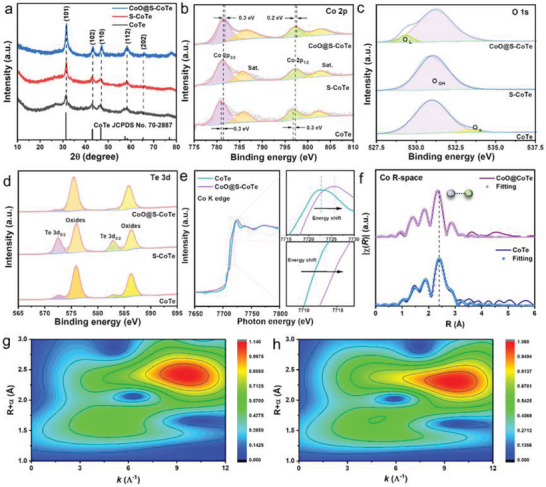
a) XRD patterns of CoTe, S‐CoTe and CoO@S‐CoTe. b) Co 2p XPS spectra, c) O 1s spectra, and d) Te 3d XPS spectra of all as‐prepared catalysts. e) The Co K‐edge XANES and f) Co K‐edge Fourier transform EXANES spectra of CoTe and CoO@S‐CoTe. g) and h) The wavelet transforms of CoTe and CoO@S‐CoTe.

The X‐ray diffraction (XRD) patterns reveal that both S‐CoTe and CoO@S‐CoTe possess the same crystalline structure that can be assigned to a hexagonal phase (PDF# 70–2887) of CoTe (**Figure** **2**a). The results are consistent with the lattice fringes of 0.285 nm corresponding to CoTe (101) plane in the HRTEM images of CoTe and S‐CoTe (Figures S8 and S9, Supporting Information). X‐ray photoelectron spectroscopy (XPS) was conducted to investigate the valence states of different elements in the catalysts (Figure S10, Supporting Information). In the core‐level Co 2p spectrum of CoTe, two pronounced peaks at 780.98 eV and 796.98 eV are assigned to Co 2p3/2 and Co 2p1/2, respectively, accompanied by two satellite peaks.^[^ ^14^ ^]^ Compared with CoTe and S‐CoTe, the deconvoluted Co 2p3/2 peak of CoO@S‐CoTe is upshifted by 0.3and 0.6 eV, respectively, verifying an increased oxidation state (Figure 2b). In the O 2p spectrum of CoO@S‐CoTe (Figure 2c), the peak centered at 533.48 eV belong to the adsorbed oxygen of CoTe disappears,^[^ ^15^ ^]^ but the characteristic peak of lattice oxygen with the peak at 529.38 eV appears.^[^ ^16^ ^]^ As shown in Figure 2d, the ratio of telluride oxides decreases after S doping but increases after oxygen plasma bombardment.^[^ ^17^ ^]^ The exact S and CoO content in CoO@S‐CoTe can be calculated EDS and XPS data, which is shown in Table S1 (Supporting Information). The fine electronic structures of CoO@S‐CoTe and reference samples were characterized by synchrotron based X­ray absorption fine structure (XAFS) at Co K­edge in Figure 2e–g, including XANES, Fourier and Wavelet transformed (FT­ and WT­^[^ ^18^ ^]^) EXAFS results (Figure S11, Supporting Information). Figure 2e of the normalized spectra of XANES shows a redshift at the absorption edge of CoO@S‐CoTe compared to CoTe, with a general shift of its white line toward higher energies, which demonstrates the valence of Co has increased after the introduction of heteroatoms and the formation of the heterojunction. Figure 2f shows the R space diagram of Co. The strongest peak observed is attributed to the single scattering of Co—Te, and the other peak is the single scattering of Co—O. It can be seen that the Co—Te bond length of CoO@S‐CoTe is shorter than that of CoTe, which could be due to the electronegativity of S stronger than those of Co and Te, making it attract surrounding atoms and induce the bonding reconstruction.^[^ ^5^ ^]^ As shown in Figure 2g, the wavelet transform maximum intensity for CoTe is ≈9.8 Å^−1^, which represents the coordination shell of Co—Te. For CoO@S‐CoTe, two maximum intensities at ≈9.0 Å^−1^ and 9.8 Å^−1^ can be identified (Figure 2h). Among them, the former results from the coordination shell of Co—Te, which also confirm the formation of Co—O structure.

**Figure 3:**
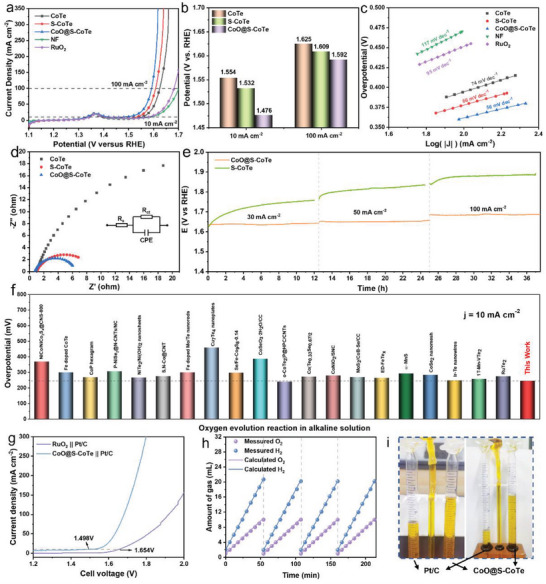
Electrochemical OER performance. a) LSV curves of CoTe, S‐CoTe, and CoO@S‐CoTe. RuO_2_ was used as reference. The measurements were conducted in 1.0 m KOH. b) Potentials at 10 mA cm^−2^ and 100 mA cm^−2^ for CoTe, S‐CoTe, and CoO@S‐CoTe. c) Tafel slopes of CoTe, S‐CoTe, and CoO@S‐CoTe. d) Electrochemical impedance spectroscopy of CoTe, S‐CoTe, and CoO@S‐CoTe. e) Comparison of the overpotential of CoO@S‐CoTe with other electrocatalysts at current densities of 10 mA cm^−2^. f) Time‐dependent potential curves of CoO@S‐CoTe under different static current density of 10, 50, and 100 mA cm^−2^. g) Comparison of the overall water splitting activities between Pt/C || CoO@S‐CoTe and Pt/C || RuO_2_ in 1.0 m KOH. h) Amount of gas collected and calculated for Pt/C || CoO@S‐CoTe. i) H_2_, O_2_ amount for Pt/C || CoO@S‐CoTe at a fixed current density of 50 mA cm^−2^ and optical picture of the measured setup of the Hoffman apparatus.

The OER performance of CoO@S‐CoTe was evaluated by the steady‐state electrochemical measurements in 1 m KOH using a standard three‐electrode system (**Figure** **3**). Remarkably, CoO@S‐CoTe requires only 246 mV of overpotential for reaching a current density of 10 mA cm^−2^, which is ≈78 mV lower than the CoTe and 56 mV lower than S—CoTe. The overpotentials at a higher current density, like 100 mA cm^−2^, for different catalysts are also compared. Similarly, CoO@S‐CoTe shows the smallest overpotential value of 362 mV at 100 mA cm^−2^ (Figure 3a,b). As NiFe LDH is acknowledged as a highly‐efficient OER electrocatalyst, we also tested its OER performance as reference. Obviously, CoO@S‐CoTe outperforms NiFe LDH in OER electrocatalysis (Figure S12, Supporting Information). To further confirm the enhancing effect caused by incorporation of suitable oxygen and sulfur species in CoTe, the LSV curves of S‐CoTe and CoO@CoTe are also provided for comparison (Figure S13, Supporting Information), which show a more favorable OER activity for CoO@S‐CoTe than that of S—CoTe and CoO@CoTe. This suggests there should be a beneficial synergy for the oxygen and sulfur species toward improving the OER performance of CoTe. Further experiments were conducted to reveal the enhancing mechanism originated from dual integrated oxygen and sulfur in CoTe. The cobalt oxide layer effect on the OER activity of CoO@S‐CoTe were first investigated by changing the treatment time of oxygen plasma (1 min, 5 min, 15 min, 30 min). Polarization curves show CoO@S‐CoTe‐15 min has the best electrochemical activity (Figure S14, Supporting Information). The Co 2p spectra (Figure S15, Supporting Information) present that the oxidation state of Co increases with prolonging oxygen plasma bombardment time. According to the XPS results, reasonable speculation can be obtained that moderate rise of Co oxidation state promotes the OER activity, while over oxidation compromises the promotion effect. Second, the influence of S content in CoO@S‐CoTe on the OER activity was studied by changing the quality of S powder. As observed in Figure S16 (Supporting Information), CoO@S‐10mg‐CoTe exhibits the highest OER activity, indicating suitable S content is another key point to attain the optimal catalysts. Tafel plots provide further insights into the OER kinetics (Figure 3c). CoO@S‐CoTe has the lowest Tafel slope value (56 mV dec^−1^) as compared with other reference samples, such as S—CoTe (66 mV dec^−1^) and CoTe (74 mV dec^−1^), implying the fastest reaction kinetics upon CoO@S‐CoTe to suffer a rapid oxygen evolution. The charge transfer resistances (*R*ct) of the catalysts were also evaluated by electrochemical impedance spectroscopy (EIS). As shown in Figure 3d, CoO@S‐CoTe displays the smallest Nyquist semicircle diameter, suggesting its rapid electron transfer rate at the catalysts/electrolyte interface.

To gain more insight into the origin of the enhanced OER performance of CoO@S‐CoTe, we performed electrochemical double‐layer capacitance (*C*_dl_) measurements based on the CV curves at different scan rates (Figure S17, Supporting Information), which presented a linear correlation with the actual electrochemical active surface area (ECSA) for electrocatalysts. The calculated C_dl_ value of CoO@S‐CoTe (13.9 mF cm^−2^) is higher than other control samples (8.95 mF cm^−2^ for S—CoTe, 7.90 mF cm^−2^ for CoO@CoTe, 4.50 mF cm^−2^ for CoTe) (Figure S17a–e, Supporting Information). This indicates that dual integration of oxygen and sulfur on CoTe contributes to increase the active site numbers. After normalization by ECSA estimated from *C*_dl_, the ECSA‐based specific activity can be obtained (Figure S17f, Supporting Information). Notably, we can see an obvious trend that such CoTe‐based materials with dual modification of oxygen and sulfur surpass other catalysts under a high potential in terms of specific activity. Moreover, the mass activity normalized by catalyst loading amount was also provided, which showed the value of 0.154 A mg_Co_^−1^ at 1.6 V for CoO@S‐CoTe, much higher than the other tested samples (Figure S18, Supporting Information). To further evaluate the intrinsic activity of these catalysts, the turnover frequency (TOF) was calculated. As shown in Figure S19 (Supporting Information), the TOF values of CoO@S‐CoTe are much higher than those of CoTe, S—CoTe, and RuO_2_ at different potential. All the results regarding specific activity, mass activity, and TOF demonstrate CoO@S‐CoTe possesses the optimal intrinsic OER activity.

In addition to the excellent electrocatalytic OER activity, long‐term stability is another key factor for practical applications. Chronopotentiometry measurement was conducted to assess the durability of CoO@S‐CoTe and S‐CoTe (Figure 3e). As expected, no significant decline was discerned after 36 h continuous working at different current densities for CoO@S‐CoTe, evidencing the fact that the catalyst is stable for OER electrocatalysis. In comparison, we also tested the working stability of S—CoTe sample and found that the potential to sustain specific current density increased quite fast with continuous chronopotentiometry test, demonstrating that the robustness of CoO@S‐CoTe during OER electrocatalysis should stem from the protection effect of surface cobalt oxide layer. Electron microscopy characterizations (SEM, TEM, and HRTEM) reveal that CoO@S‐CoTe still maintains its initial morphology and structure after the OER stability test (Figures S20–S23, Supporting Information). Moreover, near‐zero change in Co K‐edge XANES and XPS spectra is observed for CoO@S‐CoTe before and after OER durability measurement (Figures S24 and S25, Supporting Information). Notably, without the protection of surface CoO, S—CoTe can be easily reconstructed and oxidized to form high‐valence Co species (Co^3+^) (Figure S26, Supporting Information), agreeing well with previous literatures on chalcogenides‐based OER pre‐catalysts.^[^ ^19^ ^]^ Such high OER activity of our prepared CoO@S‐CoTe has outperformed other reported telluride‐based electrocatalysts and can be comparable to those of most advanced Ni‐foam (NF) supported catalysts developed recently (Figure 3f; Tables S2 and S3, Supporting Information).

To demonstrate the superiority of CoO@S‐CoTe in overall water splitting, a two‐electrode configuration electrolyzer (CoO@S‐CoTe||Pt/C) was assembled by using CoO@S‐CoTe and Pt/C as anode and cathode, respectively. Benchmark catalysts, RuO_2_ paired with Pt/C (RuO_2_||Pt/C), were also tested under the same condition for comparison. From Figure 3g, it can be found that the cell voltage to afford current density of 10 mA cm^−2^ is only 1.498 V, much better than commercial RuO_2_||Pt/C (1.654 V). CoO@S‐CoTe||Pt/C only requires The water‐splitting performance of CoO@S‐CoTe||Pt/C was much better than commercial RuO_2_||Pt/C (Figure 3g). Noteworthily, CoO@S‐CoTe||Pt/C can reach as high as 300 mA cm^−2^ at a low cell voltage of less than 1.8 V, well meeting the demand of industrial water splitting application. By using a Hoffman water electrolyzer, CoO@S‐CoTe||Pt/C displays a high Faradaic efficiency close to 100% for both HER and OER, with a volume ratio of 2:1 of hydrogen to oxygen (Figure 3h and i). Meanwhile, CoO@S‐CoTe||Pt/C shows a promising long‐term stability. After continuous working at 10 and 100 mA cm^−2^ for 24 h, no obvious increase for cell voltage can be observed (Figures S27 and S28, Supporting Information). These results confirm CoO@S‐CoTe is quite suitable as ideal anode candidate catalyst for practical H_2_ production via water splitting system.

**Figure 4:**
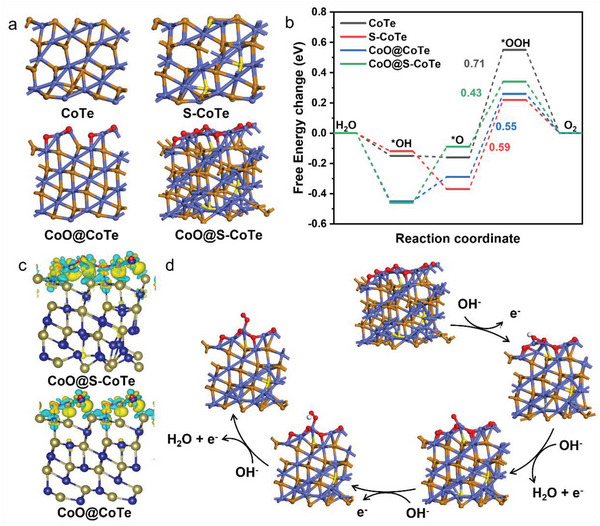
a). Schematic structure model of CoTe, S‐CoTe, CoO@CoTe, and CoO@S‐CoTe. (atoms with blue, orange, yellow, and red colors represent Co, Te, S, and O atoms, respectively). b) Free energy diagrams at 1.23 V for the OER process on CoTe, S‐CoTe, CoO@CoTe, and CoO@S‐CoTe. c) Charge density redistributions of CoO@CoTe and CoO@S‐CoTe d) Proposed OER mechanism of CoO@S‐CoTe.

To fundamentally decode the function of oxygen and sulfur species on the OER activity of CoO@S‐CoTe, DFT calculations were performed to study the charge distribution and Gibbs free‐energy of elementary steps (**Figure** **4**). In consistent with our experimental results, four theoretical models in Figure 4a, including CoTe, S—CoTe, CoO@CoTe, and CoO@S‐CoTe, were constructed. Figure 4b and Figure S29 (Supporting Information) depict the energy diagrams at 1.23 V and 0 V. As seen obviously, conversion of O* to OOH*, the third step of OER process, is the rate‐determining step (RDS) for all the models. The ∆*G*_O*→OOH*_ value of S—CoTe (0.59 eV) and CoO@CoTe (0.55 eV) is smaller than that of CoTe (0.71 eV), demonstrating the positive effect of S doping and surface oxidation in decreasing the OER energy barrier. Note that CoO@S‐CoTe has the minimum ∆*G*_O*→OOH*_ value of 0.43 eV among the four models, which indicates a stronger binding strength between intermediate O* atom and exposed Co site of top CoO layer within CoO@S‐CoTe model.^[^ ^20^ ^]^ The above theoretical analysis proves that the activity promotion level in CoTe can be further improved by introducing synergetic oxygen and sulfur regulation. Table S4 (Supporting Information) lists the adsorption energy of all OER intermediates and the corresponding contour volcano plot of OER overpotential is displayed in Figure S30 (Supporting Information). The descriptor position of CoO@S‐CoTe locates close to the white region, which means near‐optimal intermediates binding energy and near‐minimum OER overpotential that can be achieved in different systems of our work. Based on this contour map, we can conclude that dual integrating oxygen and sulfur modulation benefits to approach the OER activity limit of CoTe. To further unveil the activity enhancement mechanism, differential charge density distribution is calculated and displayed in Figure 4c. Obviously, doping S in CoTe can lead to more convenient charge transfer from top CoO layer to the internal Co site, which creates a consequent electron deficiency area in CoO so as to boost OER catalytic performance. Figure 4d and Figures S31–S33 (Supporting Information) are schematic illustrations of the proposed OER mechanism for various models. The well‐matched experimental and theoretical results verify that doping S in CoTe lattice together with formation of a proper surface CoO can synergistically modulate the OER performance of CoTe to break its practical application bottleneck.

## 3 Conclusion

In summary, we have reported that the activation of cobalt telluride (CoTe) nanoarrays toward enhanced OER can be achieved by constructing the CoO@S‐CoTe heterostructure with dual integration of sulfur (S) doping and surface oxidization. The outstanding OER activity of CoO@S‐CoTe catalyst was evidenced by a substantially low overpotential of only 246 mV at the current density of 10 mA cm^−2^ for continuous over 12 h at larger current density of 10 mA cm^−2^ benefiting from stability of designed heterostructure, which to our knowledge outperforms the reported telluride‐based OER catalysts. Remarkably, the overall water‐splitting performance of CoO@S‐CoTe||Pt/C was superior to commercial RuO_2_||Pt/C. More importantly, the combined experimental and theoretical results reveal that the enhanced OER performance stems from the exposure of increased active sites, which can efficiently optimize charge transfer ability and electronic state. Thus, the discovery of constructing the CoO@S‐CoTe heterostructure can provide a valuable guidance to release the catalytic potential of telluride‐based OER catalysts.

## Conflict of Interest

The authors declare no conflict of interest.

## Supporting information

Supporting Information

## Data Availability

The data that support the findings of this study are available from the corresponding author upon reasonable request.
