# Supplementary material for: Dual Integrating Oxygen and Sulphur on Surface of CoTe Nanorods Triggers Enhanced Oxygen Evolution Reaction
Source: Adv Sci (Weinh). 2023 Jan 26;10(9):2206204. doi: 10.1002/advs.202206204 (PMC10037960; doi:10.1002/advs.202206204)
Supplement: Supplementary file 1 — Supporting Information [file ADVS-10-2206204-s001.pdf]

Supporting Information

**Dual integrating oxygen and sulphur on surface of CoTe nanorods triggers enhanced oxygen evolution reaction**

*Xin Wang#, Zhelin Mao#, Xin Mao#, Ximiao Hu, Feiyue Gao, Minrui Gao, Qi-Long Wu, Xiao Lyu, Aijun Du, Xiangsheng Xu*, Yi Jia*, and Lei Wang**
*# These authors contributed equally*

Prof. X. Wang, Z. Mao, X. Hu, Prof. X. Xu, Prof. Y, Jia, Prof. L, Wang
College of Chemical Engineering
Zhejiang University of Technology
Hangzhou 310032, P. R. China.

X. Mao, Prof. A. Du
School of Chemistry, Physics and Mechanical Engineering
Queensland University of Technology
Brisbane, QLD 4000, Australia

F. Gao, Prof. M. Gao
Department of Chemistry, Institute of Biomimetic Materials & Chemistry, Anhui Engineering Laboratory of Biomimetic Materials, Division of Nanomaterials & Chemistry, Hefei National Research Center for Physical Sciences at the Microscale, Institute of Energy, Hefei Comprehensive National Science Center
University of Science and Technology of China
Hefei 230026, China

Q-L, Wu
Intelligent Polymer Research Institute and ARC Centre of Excellence for Electromaterials Science, Australian Institute for Innovative Materials
University of Wollongong, Wollongong, NSW 2500, Australia

Prof. X. Lyu
School of Materials Science and Engineering
Shenyang Ligong University
Shenyang 110159, P. R. China

**1. Materials.**

Sodium tellurite ($Na_2TeO_3$) and Nafion solution (5 wt.% in alcohol and water) were purchased from Aladdin. Cobalt sulfate heptahydrate ($CoSO_4·7H_2O$), Ammonia water ($NH_3·H_2O$) and hydrazine hydrate ($N_2H_4·H_2O$), S powder comes from Sinopharm. Ruthenium dioxide ($RuO_2$), and a commercial Pt/C catalyst (20 wt.%; Pt on the black carbon) were purchased from Johnson Matthey Corporation. Milli-Q ultrapure water was used for experiments. All these chemical reagents were used without any further purification. Nickel foam (NF) (thickness: 1 mm) were used as the substrate.

**2. Synthesis**

**Synthesis of CoO@S-CoTe.** Ni foam was sonicated with the HCl solution (2 M) for 15 min, followed by sonicating in ethanol and water for 20 min, respectively. Finally, the Ni foam dried in air for using. The synthesis of CoTe materials referred to literature with modification. The $CoSO_4·7H_2O$ (0.5 mmol) was dissolved in 20 mL of deionized water. Then, 0.5 mmol $Na_2TeO_3$ was added into above solution, and a precipitation of $CoTeO_3$ appeared emerged immediately. After stirring 20 min, 2 mL $NH_3$ $H_2O$ and 0.4 mL $N_2H_4·H_2O$ were added into the mixture. Until the precursor solution was blended evenly, the solution was transferred into 50 mL Teflon-lined autoclave and a piece of cleaned Ni foam with 1 × 4 cm was put inside it. Then, the autoclave was sealed, and heated at 140 °C for 5 h. After the reaction was completed, the system was cooled to room temperature. Black CoTe products were grown on Ni foam. CoTe catalysts were washed with deionized water and ethanol, and then dried at

60 °C. 10mg s powder and 1 * 2cm CoTe were annealed, then heated at 5 °C/min and annealed at 300 °C for 2 hours in Ar atmosphere. CoO@S-CoTe was prepared by bombarding S-CoTe with oxygen plasma.

$$2CoSO_4 + 2Na_2TeO_3 + 3N_2H_4 \xrightarrow{140\ ^\circ C} 2Co + 2Te + 3N_2\uparrow + 2Na_2SO_4 + 6H_2O$$

$$Co + Te \longrightarrow CoTe$$

$$CoTe + S_{(S)} \longrightarrow S\text{-}CoTe + S_{(g)}$$

$$S\text{-}CoTe + O_2 \longrightarrow CoO@S\text{-}CoTe$$

**3. Characterization**

Scanning electron microscope (SEM) images were obtained on a Zeiss Geminisem 500 microscope at 5 kV. Transmission electron microscope (TEM), selected area electron diffraction (SAED), high-resolution TEM (HR-TEM), high-angle annular dark field-scanning transmission electron microscopy (HAADF-STEM) and energy dispersive X-ray spectroscopy (EDX) mapping images were acquired on a JEOL JEM-2100F 300 kV. X-ray diffraction (XRD) patterns were recorded with an Rigaku Ultima IV operated at 40 kV with Cu Kα radiation (λ = 0.15406 nm) in the theta range is from 10° to 80° and the scan rate is 10°/min. X-ray photoelectron spectroscopy (XPS) was performed on a Thermo Scientific K-Alpha instrument operated at 12 kV with Al Ka radiation. The Co K-edges XANES spectra of catalysts were measured at the photoemission end-station at beamline 4B9A in the BSRF, Beijing, China.

**4. Electrochemical measurements**

The electrochemical measurements were performed using a three-electrode system by a CHI 660D electrochemistry station. A carbon rod was used as the counter electrode, a Ag/AgCl electrode (saturated KCl-filled) was used as the reference electrode and integrated 3D electrode of CoTe were severed as working electrode. The preparation of commercial Pt/C and $RuO_2$ electrodes was shown as follows. 1.53 mg Pt/C and 1.51mg $RuO_2$, 20 μL Nafon solutions were dispersed in ethanol (500 μL) by ultrasonication to make a homogeneous catalyst ink. Then, the ink was all dipped on 1*1 $cm^2$ Ni foam to achieve a mass-loading of about 1.5 mg $cm^{-2}$ for commercial samples. All electrochemical measurements were carried out in 1 M KOH solution. All potentials measured were transformed to reversible hydrogen electrode (RHE) based on Nernst equation: $E_{RHE} = E_{(vs.\ Ag/AgCl)} + 0.197\ V + 0.059\ pH$. The OER measurements were recorded by LSV at a scan rate of 5 mV $s^{-1}$. All polarization curves were corrected with 90% iR-compensation. Electrochemical specific surface area (ECSA) of the electrode was calculated by performing cyclic voltammetry (CV) cycles in an applied potential range of 1.1 ~ 1.2 V (vs. Ag/AgCl). Electrochemical impedance spectroscopy (EIS) was recorded with a frequency ranging from 0.01 to $10^5$ Hz with an AC amplitude of 5 mV at two overpotentials of 300 mV. As for the Faraday efficiency (FE) measurements, the gaseous products were collected using a Hoffman water electrolysis apparatus. The production of $H_2$ and $O_2$ was measured separately in 1 M KOH electrolyte at a current density of 50 mA $cm^{-2}$ for 213 min.

**Turnover Frequency (TOF) Calculations:**

$$\mathrm{TOF} = \frac{j \times A}{4 \times F \times n}$$

where the number 4 means 4 electrons $mol^{-1}$ of $O_2$ for OER, F is Faraday constant (96485 C $mol^{-1}$), n is the number of moles of the Co element of CoO@S-CoTe electrode, j is Current (A) during the linear sweep measurement.

**5. DFT calculations**

All the calculations were performed by using the density functional theory (DFT) method as implemented in the Vienna Ab initio Simulation Package (VASP)[1]. The Perdew-Burke-Ernzerhof (PBE) functional of the generalized gradient approximation (GGA) was used for the calculation of electron exchange-correlation with the projector augmented wave (PAW) method[2]. Spin-polarization was also included through the calculations, and the cut-off energy of 500 eV for plain-wave basis sets was adopted. The convergence threshold was set to $10^{-5}$ eV, and $5\times10^{-3}$ eV/Å for energy and force, respectively. The weak interaction was described by DFT+D3 method using empirical correction in Grimme’s scheme[3]. At least 15 Å vacuum space was applied in the z-direction of the slab models, preventing the vertical interaction between slabs.

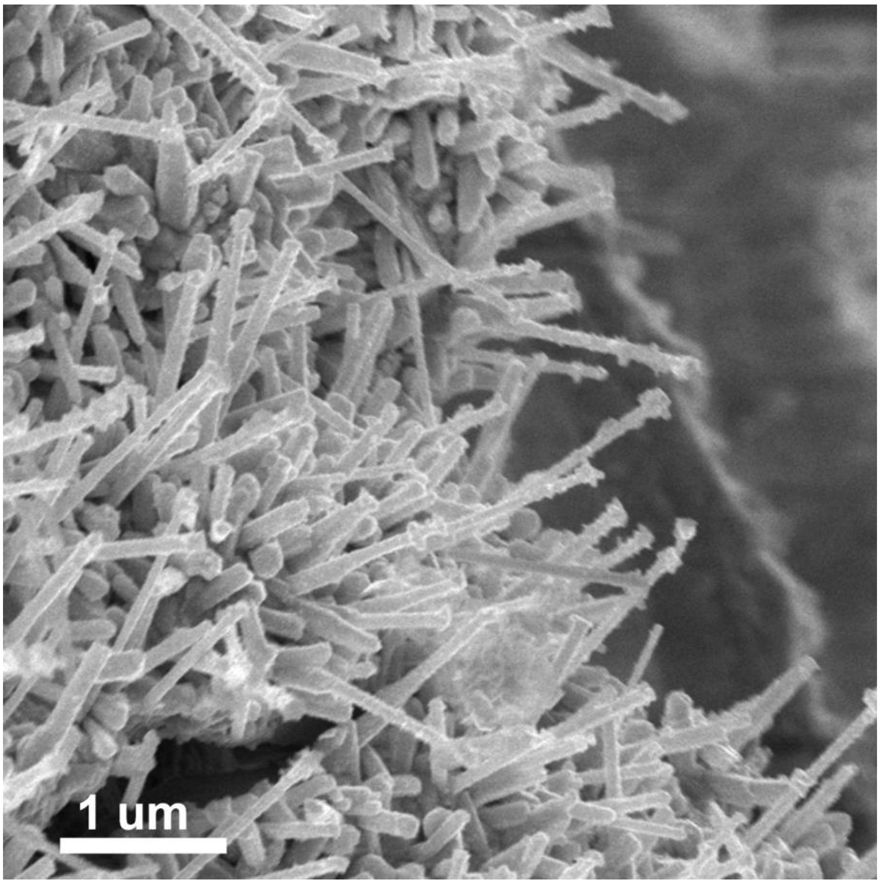


**Supplementary Figure 1**. SEM images of CoTe.

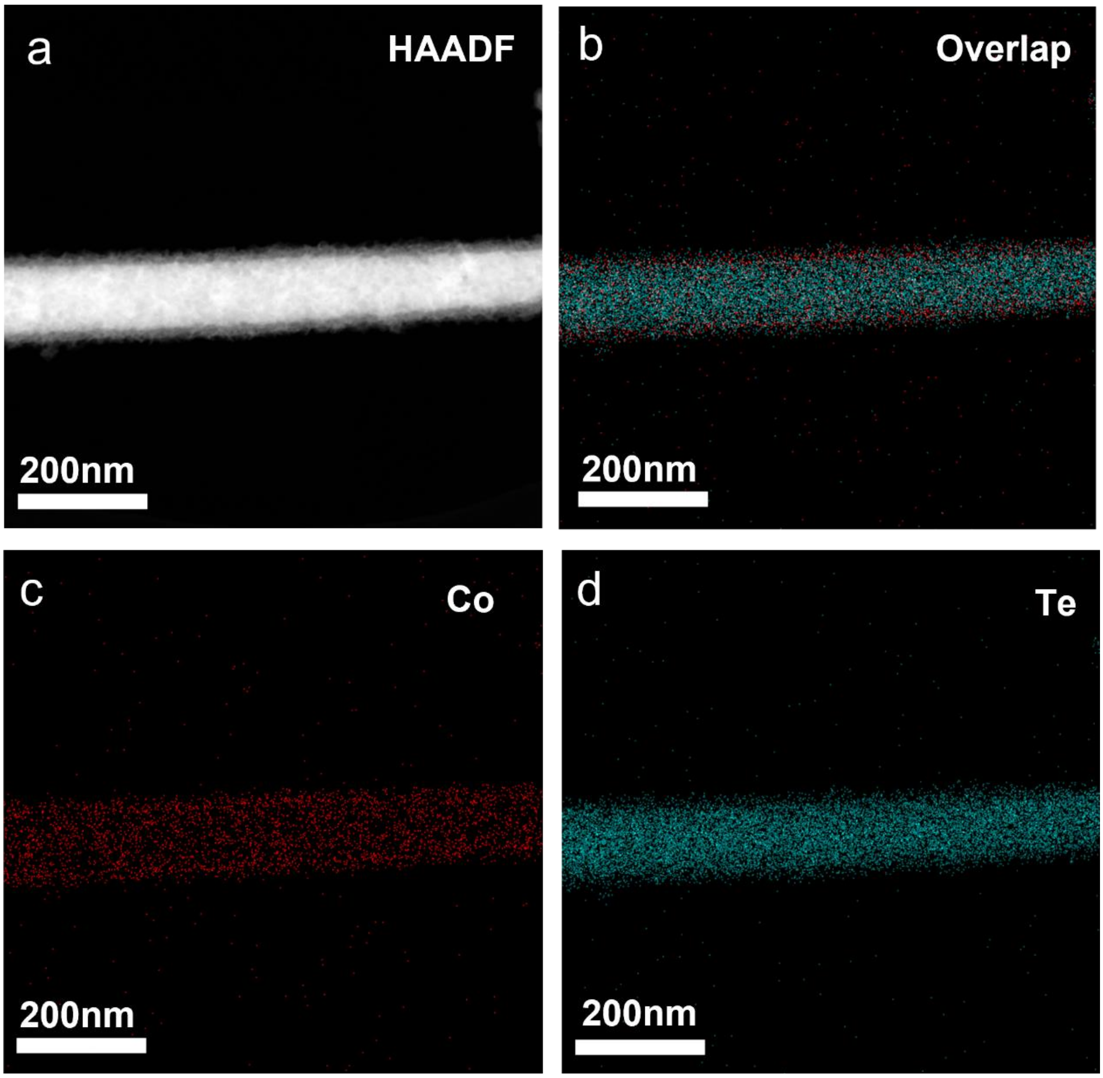


**Supplementary Figure 2**. a-c) HAADF-STEM and EDX element mapping images of CoTe.

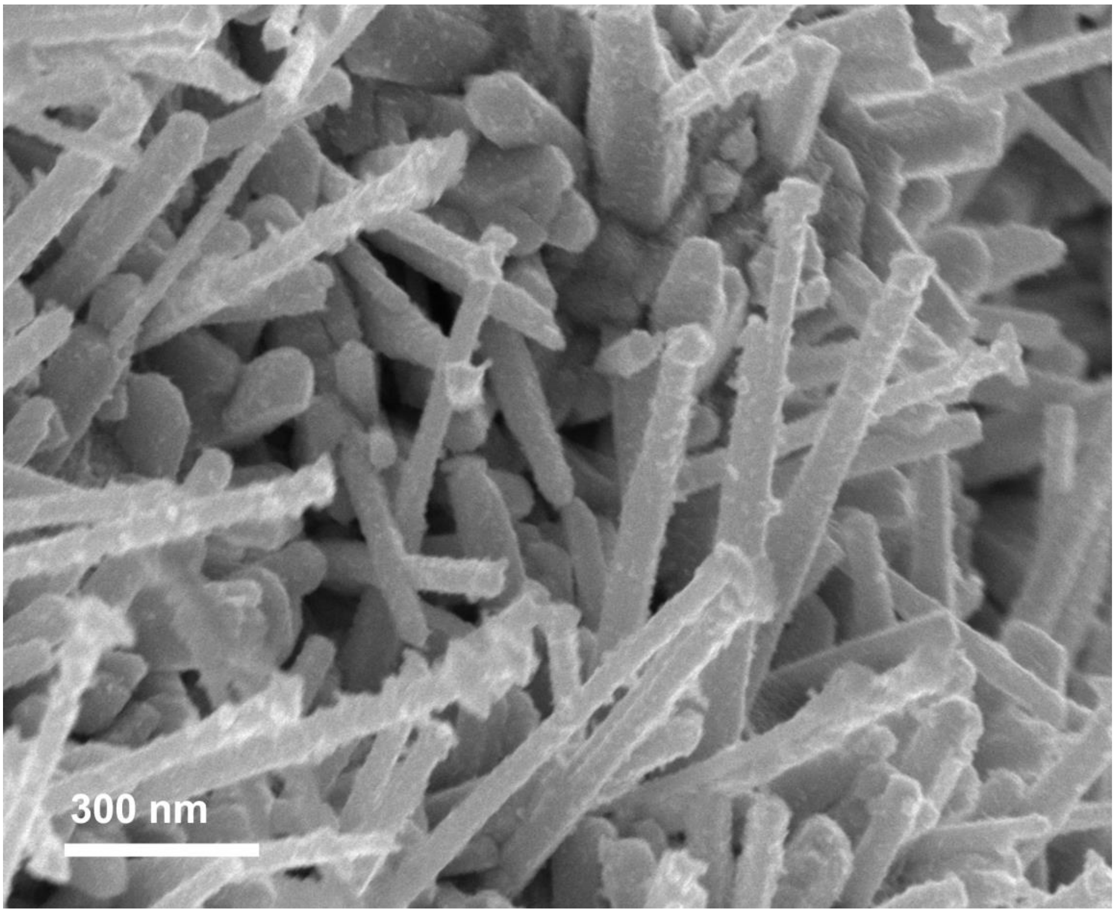


**Supplementary Figure 3**. SEM images of S- CoTe.

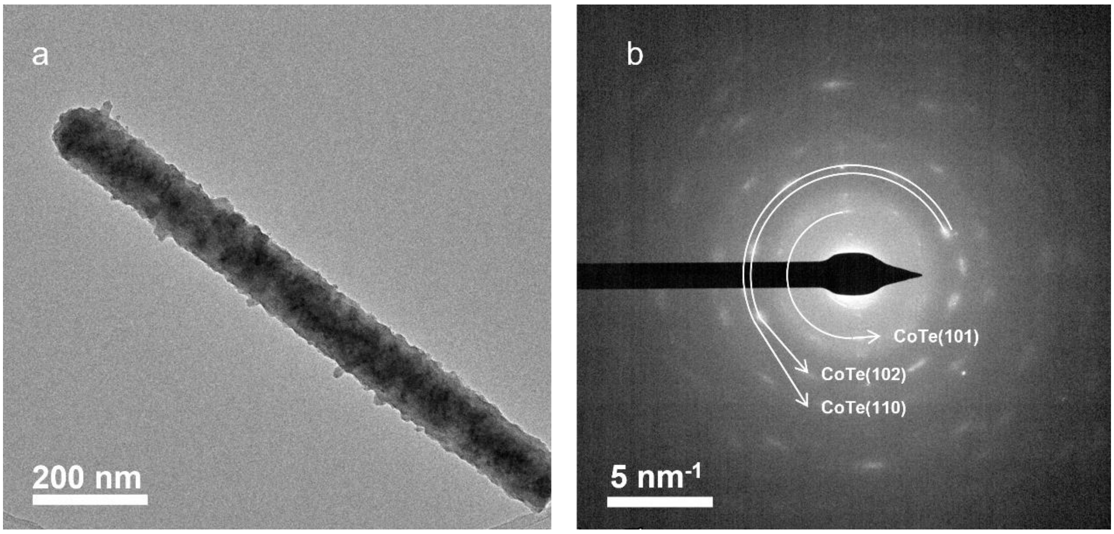


**Supplementary Figure 4**. a) and b) TEM images and corresponding SAED patterns of CoTe.

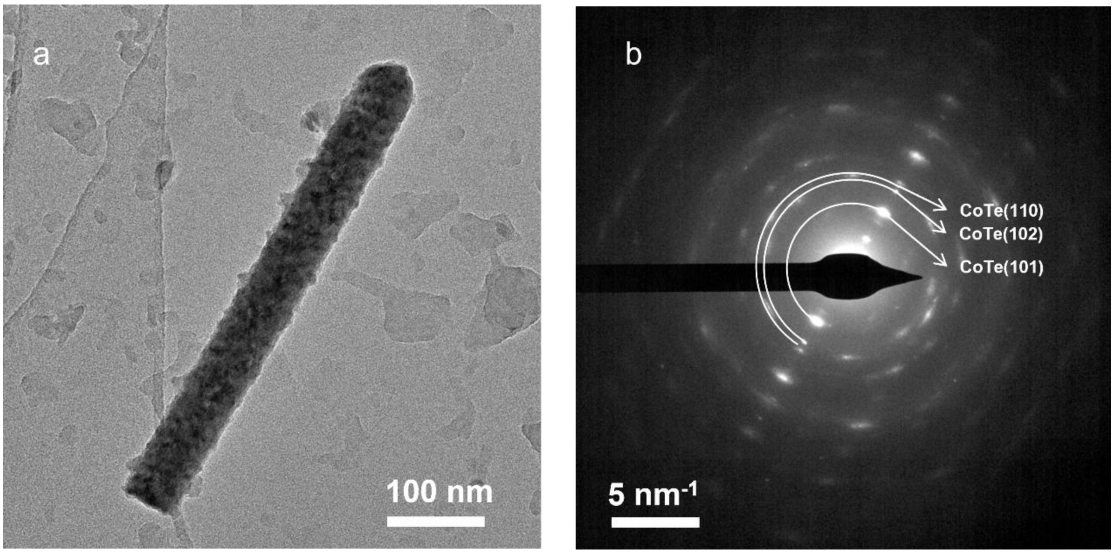


**Supplementary Figure 5**. a) and b) TEM images and corresponding SAED patterns of S-CoTe.

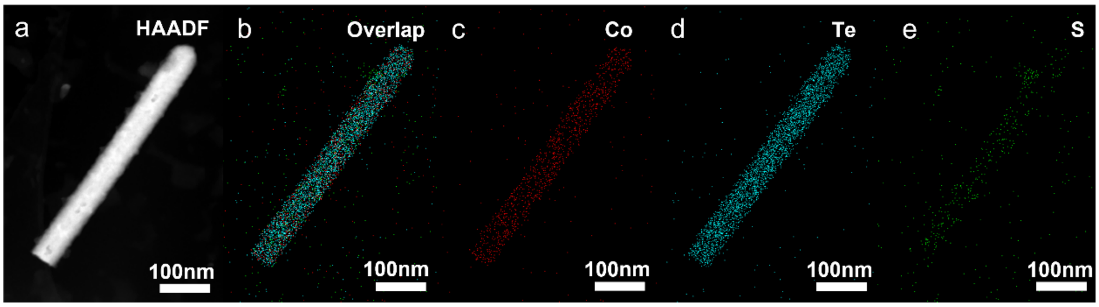


**Supplementary Figure 6**. a-e) HAADF-STEM and EDX element mapping images of S-CoTe.

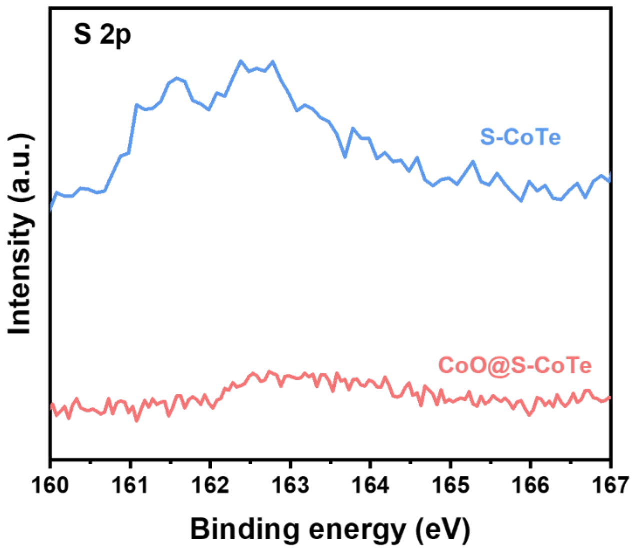


**Supplementary Figure 7**. XPS spectra on S 2p of S-CoTe and CoO@S-CoTe.

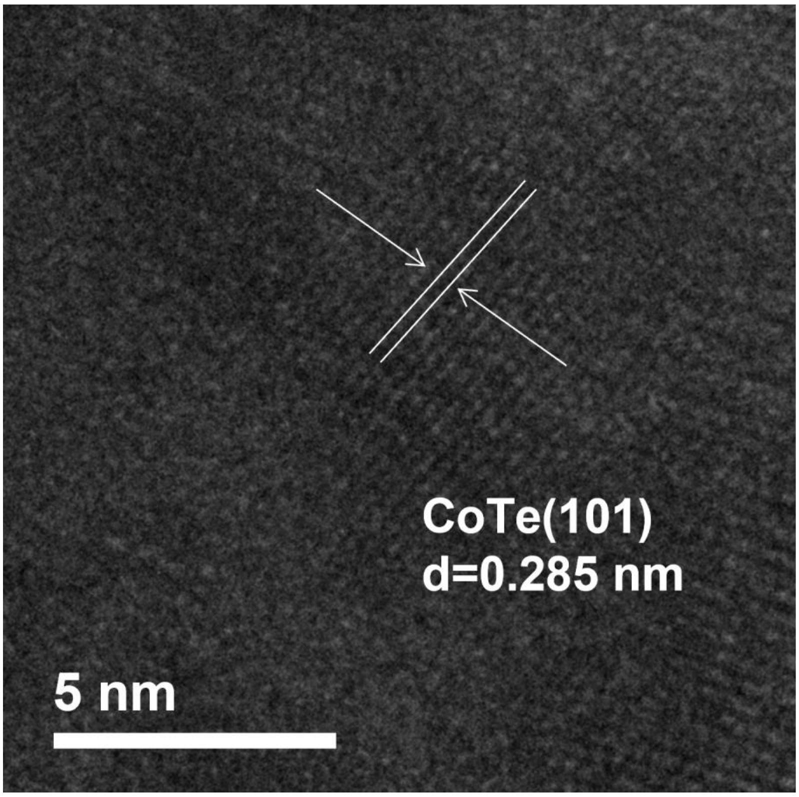


**Supplementary Figure 8**. HRTEM images of CoTe.

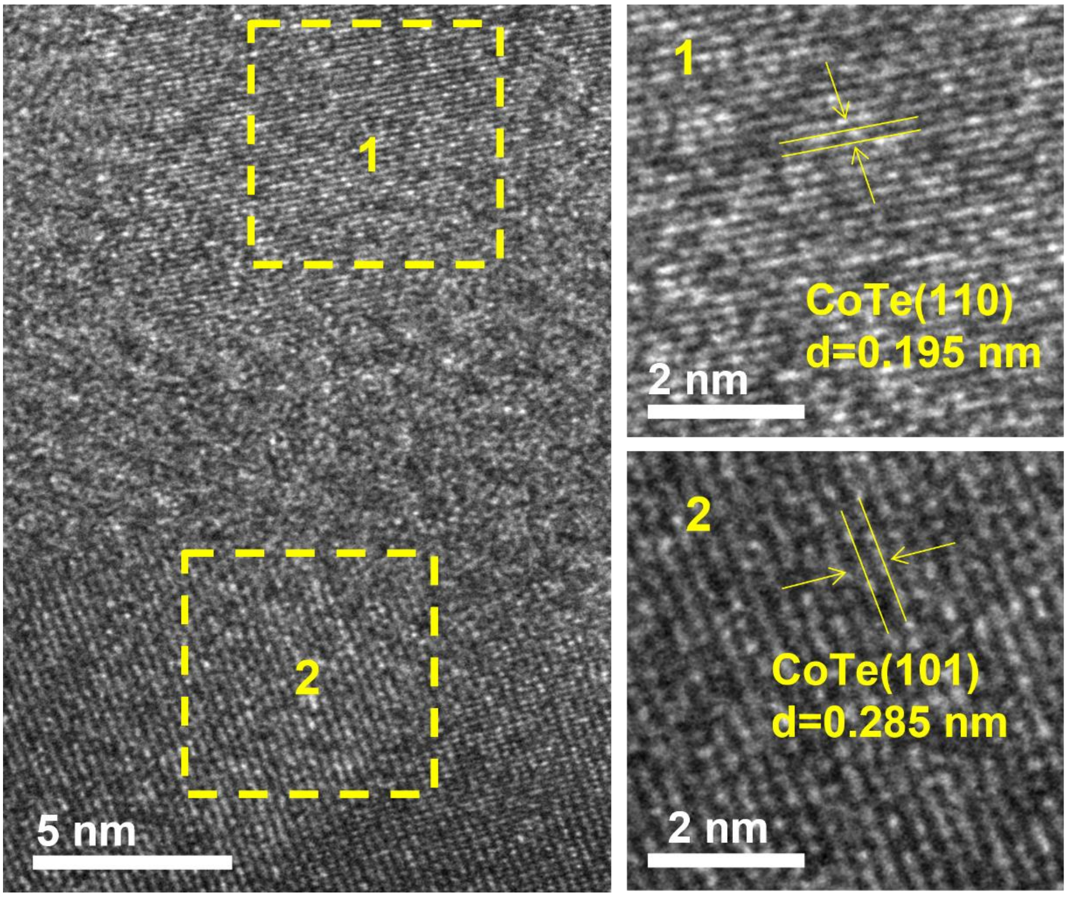


**Supplementary Figure 9**. HRTEM images of S-CoTe.

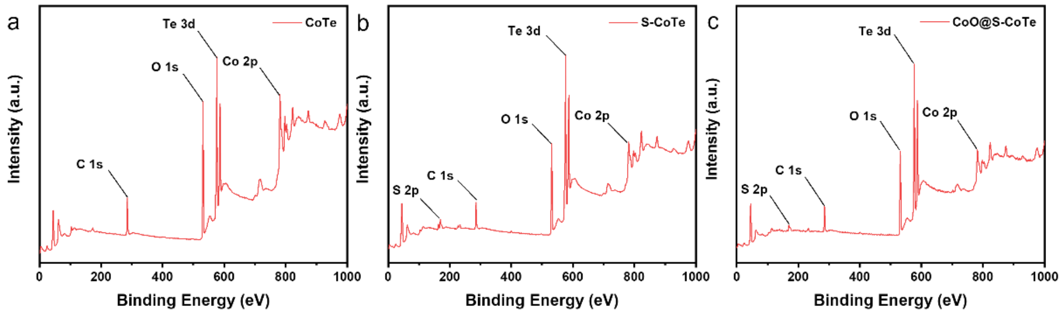


**Supplementary Figure 10**. XPS survey spectra of a) CoTe, b) S-CoTe and c) CoO@S-CoTe.

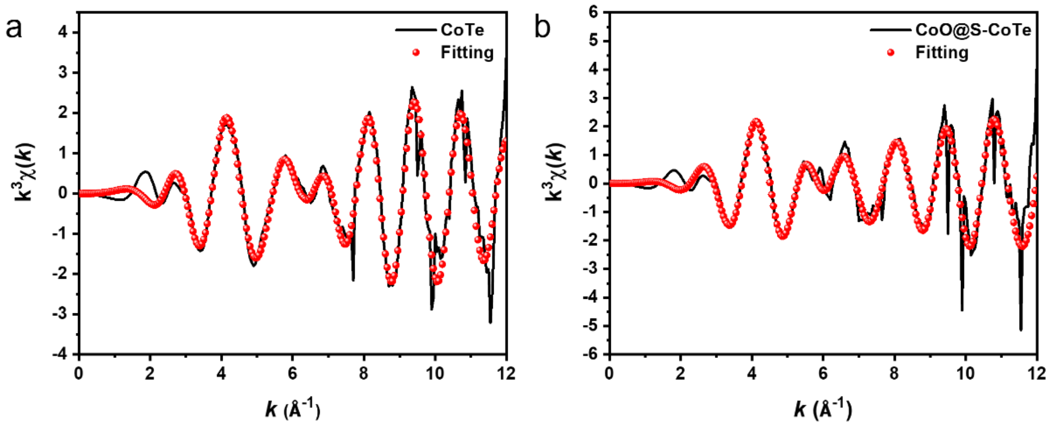


**Supplementary Figure 11**. Co K-edge extended X-ray absorption fine structure (EXAFS) spectra and corresponding fitting curves of a)CoTe and b) CoO@S-CoTe in k space.

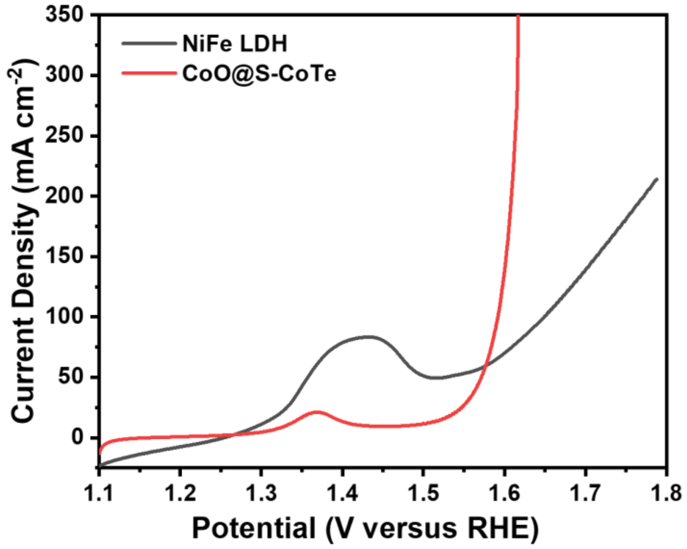


**Supplementary Figure 12**. LSV curves of CoO@S-CoTe and NiFe LDH.

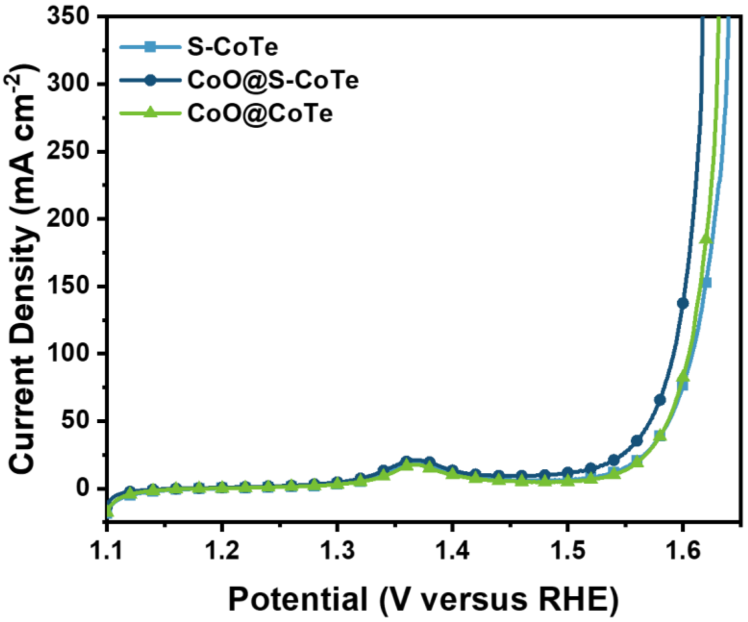


**Supplementary Figure 13**. a) LSV curves of CoO@CoTe.

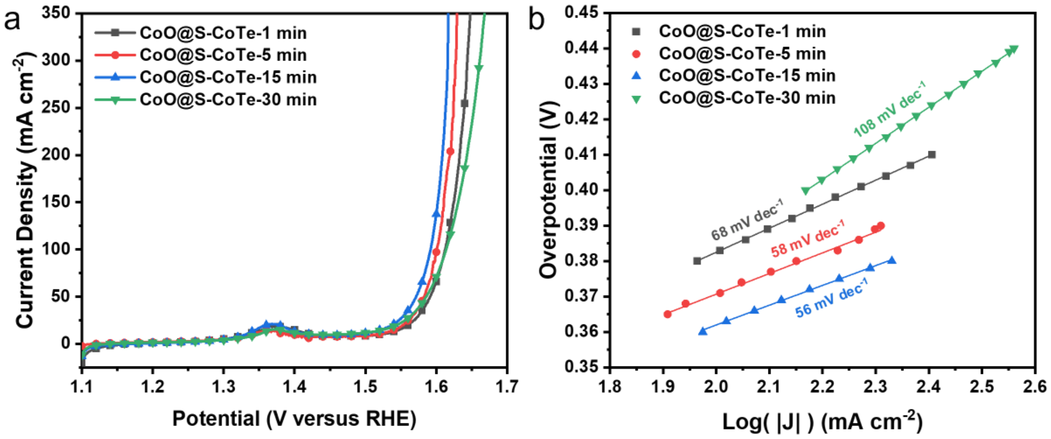


**Supplementary Figure 14**. a) LSV curves, b) Tafel slopes of CoO@S-CoTe-x min.

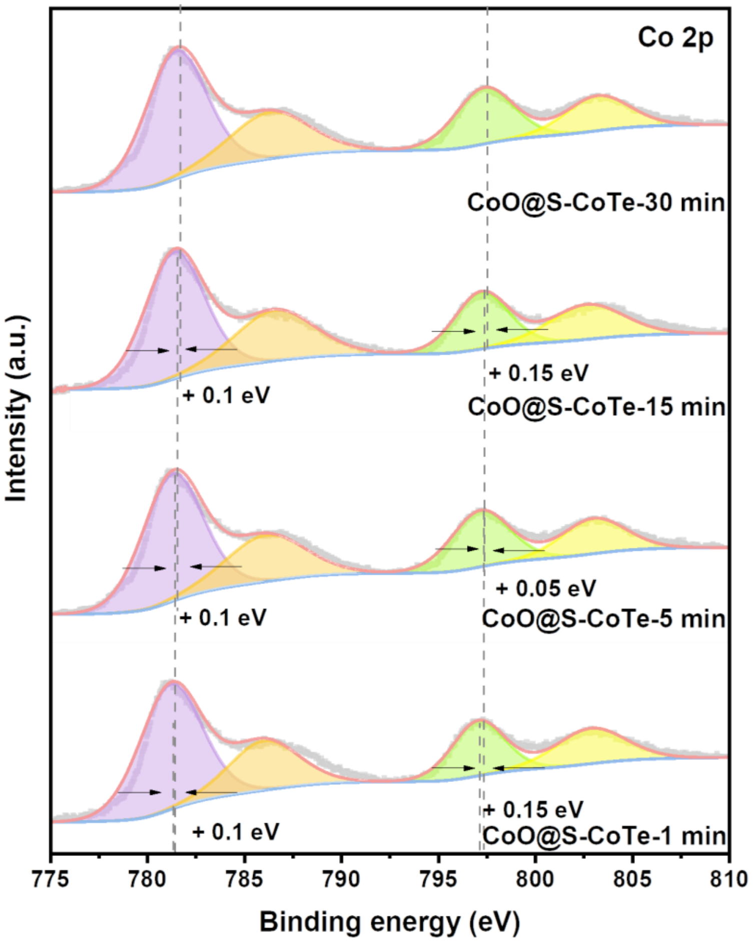


**Supplementary Figure 15**. XPS spectra of b) Co 2p for CoO@S-CoTe-x min.

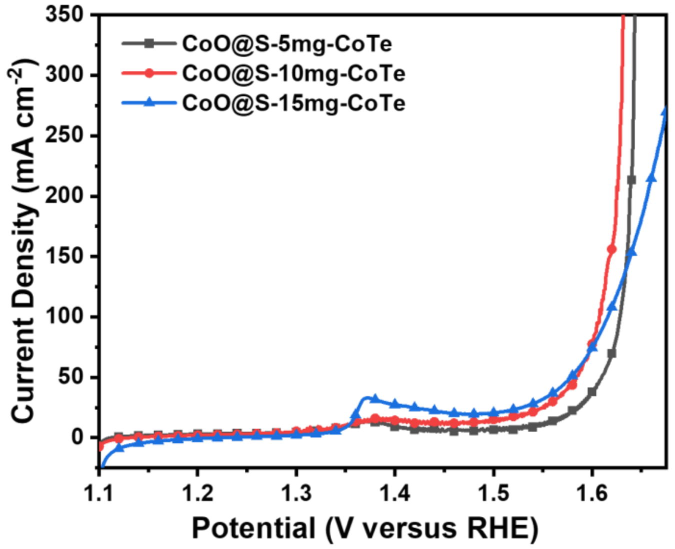


**Supplementary Figure 16**. LSV curves of CoO@S-x mg-CoTe

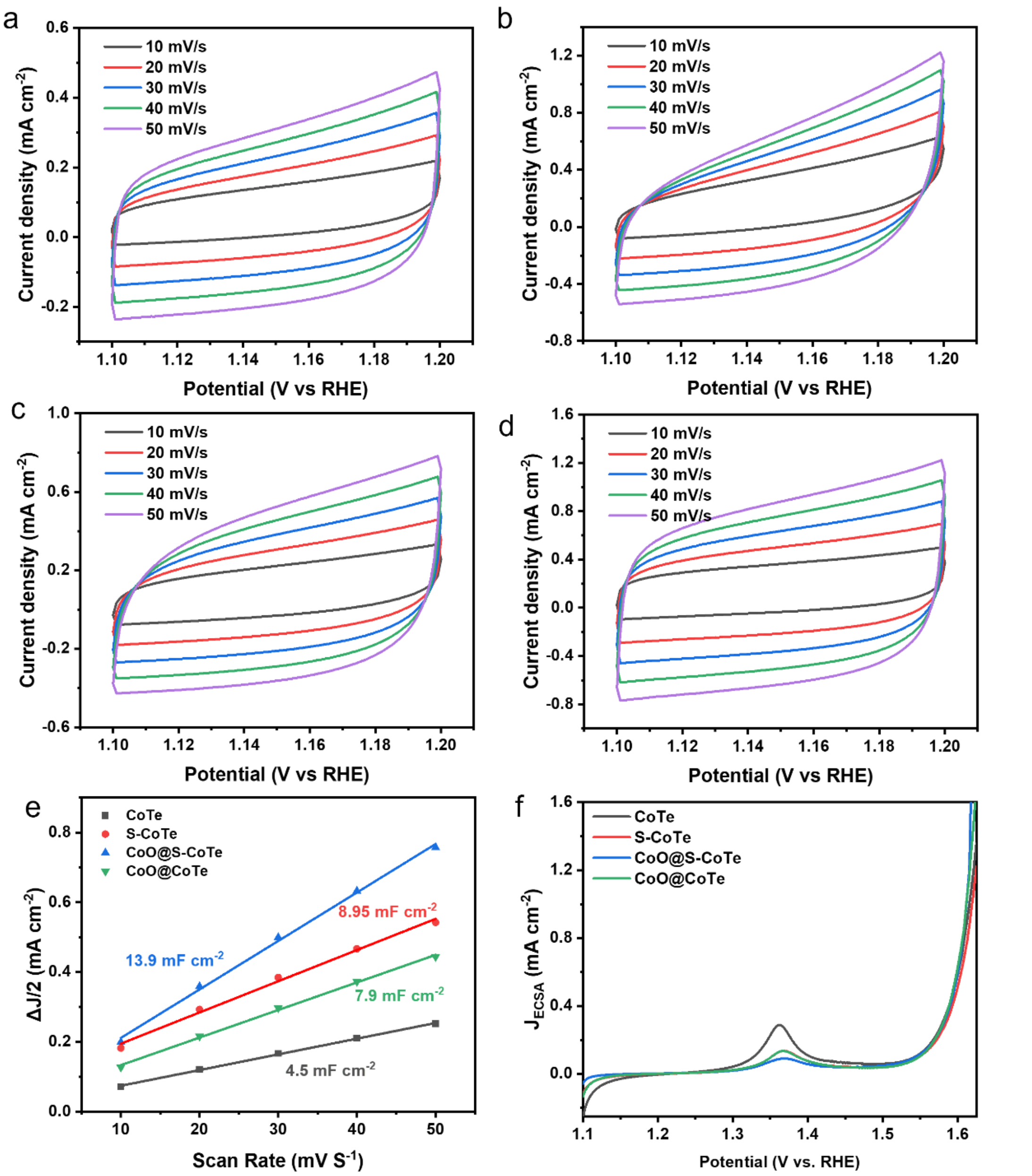


**Supplementary Figure 17**. The cyclic voltammograms (CVs) curves at different scan rates of (a) CoTe, (b) S-CoTe (c) CoO@CoTe and (d) CoO@S-CoTe ; (e) The linear fitting of scan rates with capacitive current densities ; (f) Normalized LSV curves (based on ECSA) of CoTe, S-CoTe,CoO@CoTe and CoO@S-CoTe.

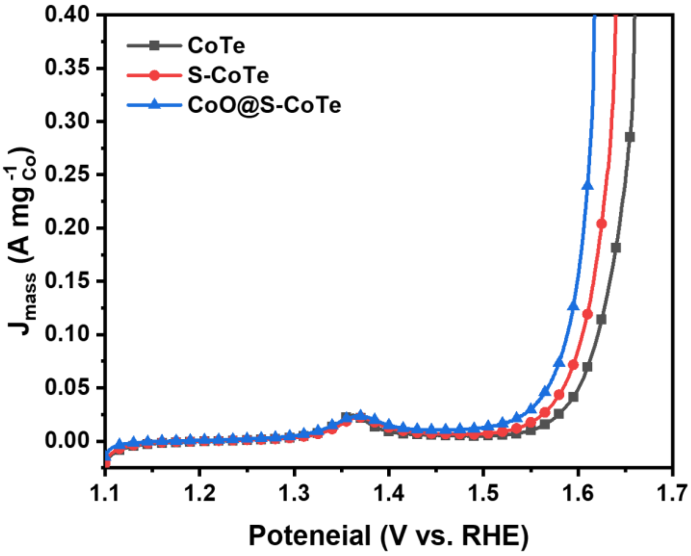


**Supplementary Figure 18**. Mass activity of CoTe, S-CoTe and CoO@S-CoTe.

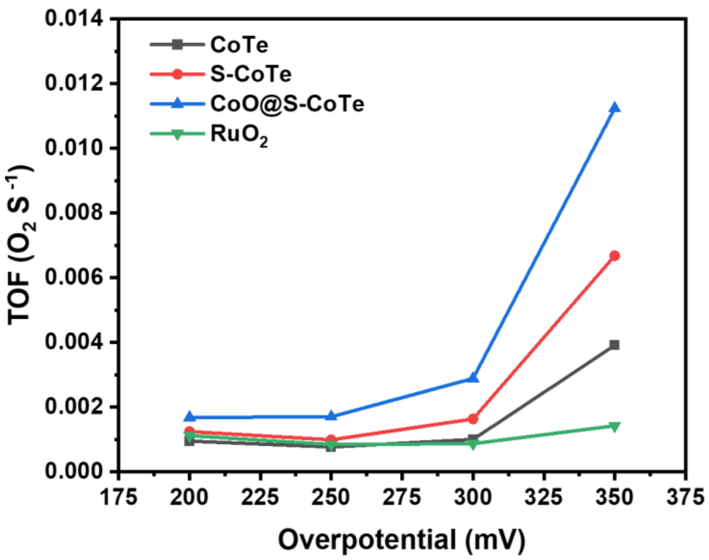


**Supplementary Figure 19**. TOF values of CoTe, S-CoTe, CoO@S-CoTe and $RuO_2$ toward OER.

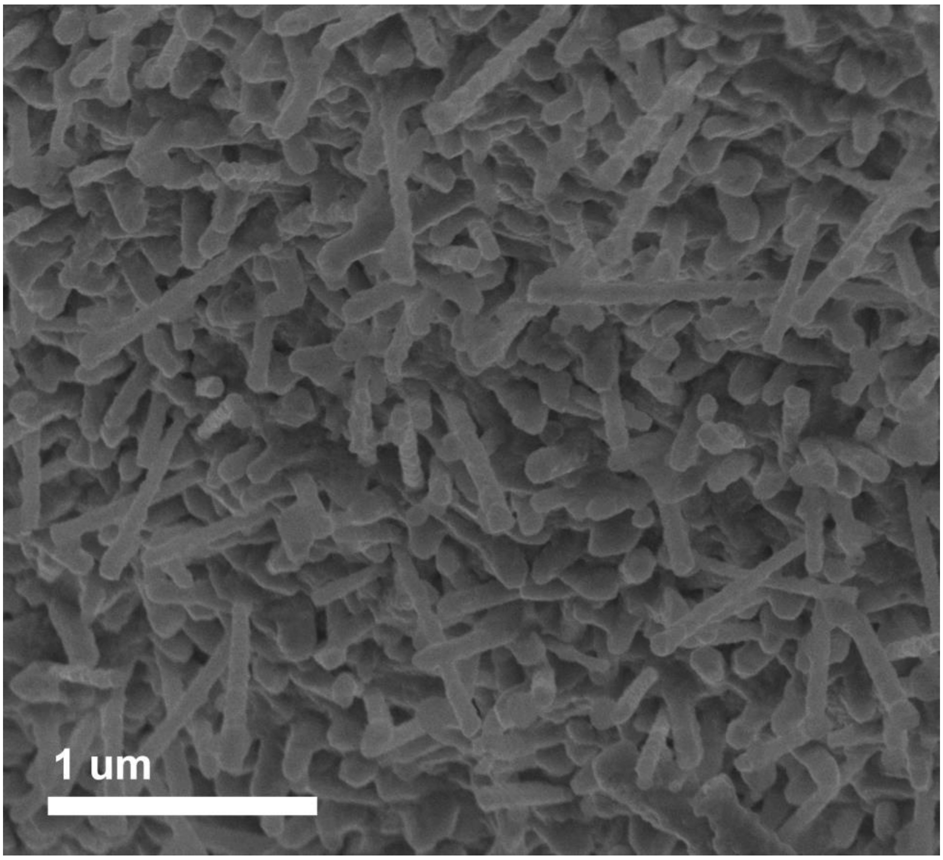


**Supplementary Figure 20**. SEM images of the CoO@S-CoTe after stability test.

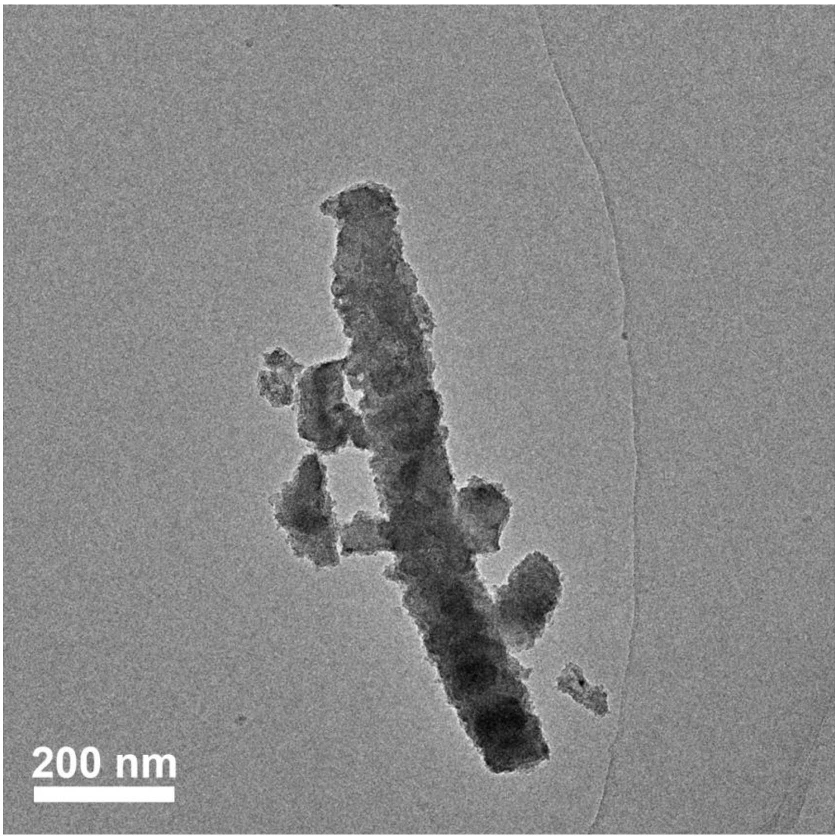


**Supplementary Figure 21**. TEM images of the CoO@S-CoTe after stability test.

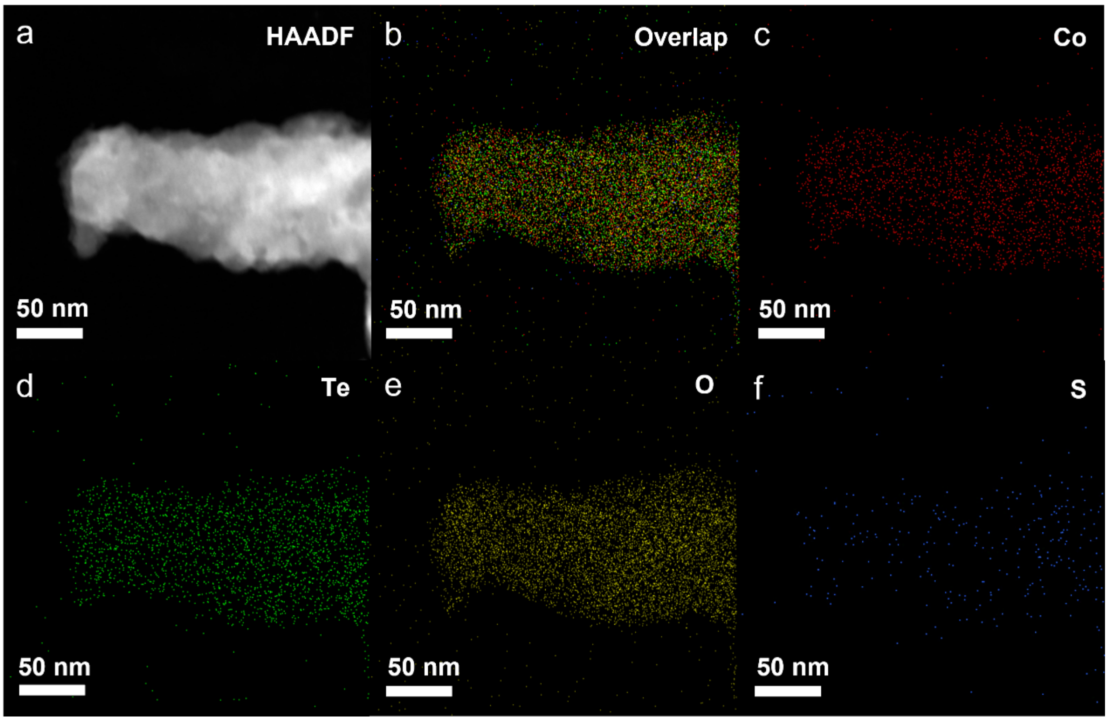


**Supplementary Figure 22**. a-f) HAADF-STEM and EDX element mapping images of CoO@S-CoTe after stability test.

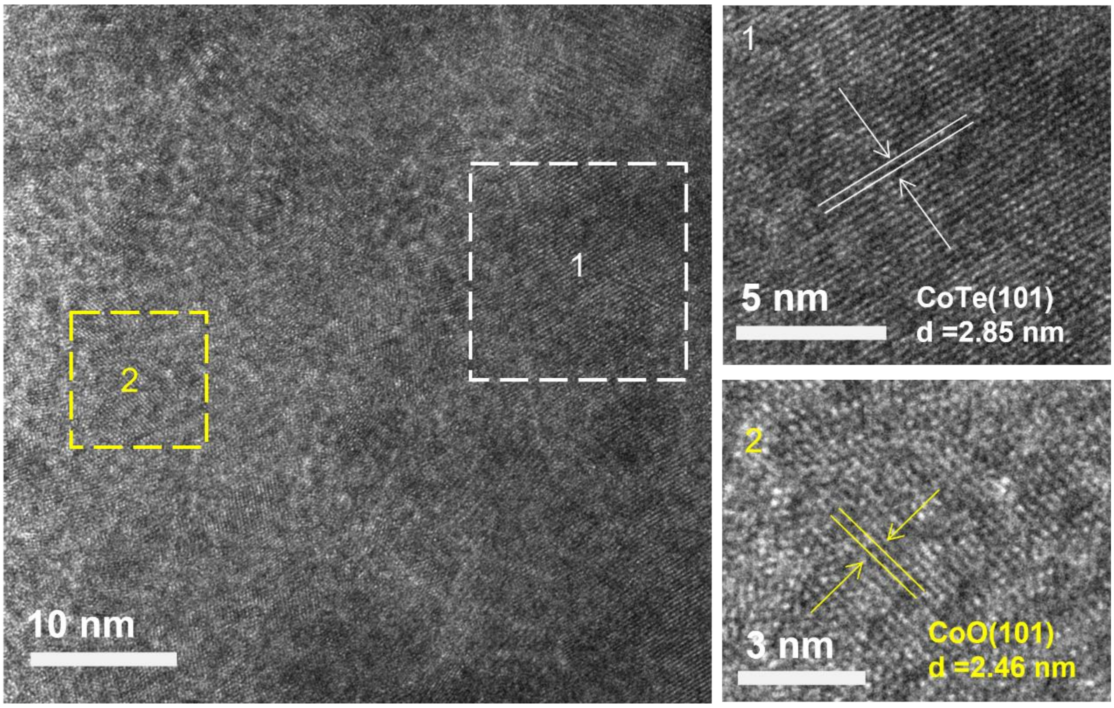


**Supplementary Figure 23**. a-c) HRTEM images of CoO@S-CoTe after stability test.

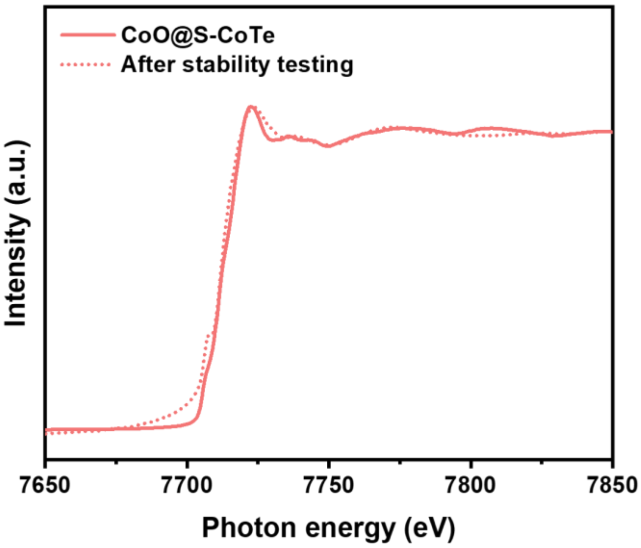


**Supplementary Figure 24**. The Co K-edge XANES of CoO@S-CoTe after stability test.

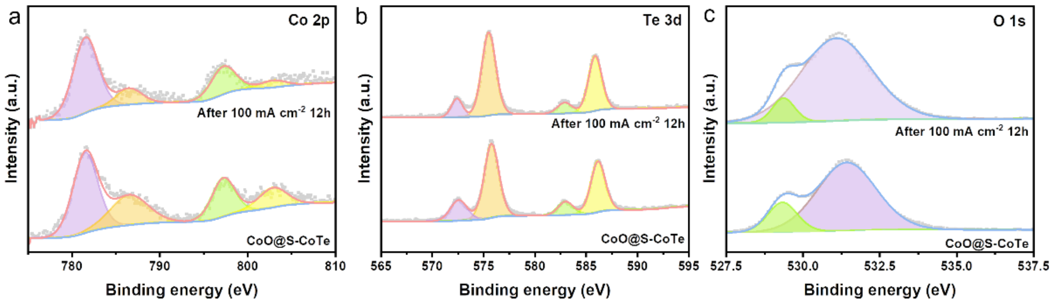


**Supplementary Figure 25**. XPS spectra of a) Co 2p, b) Te 3d and c) O 1s of CoO@S-CoTe after stability test.

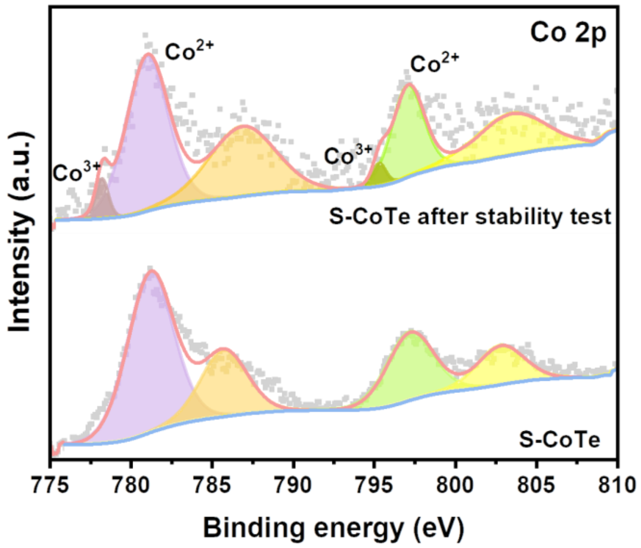


**Supplementary Figure 26**. XPS spectra of Co 2p of S-CoTe after stability test.

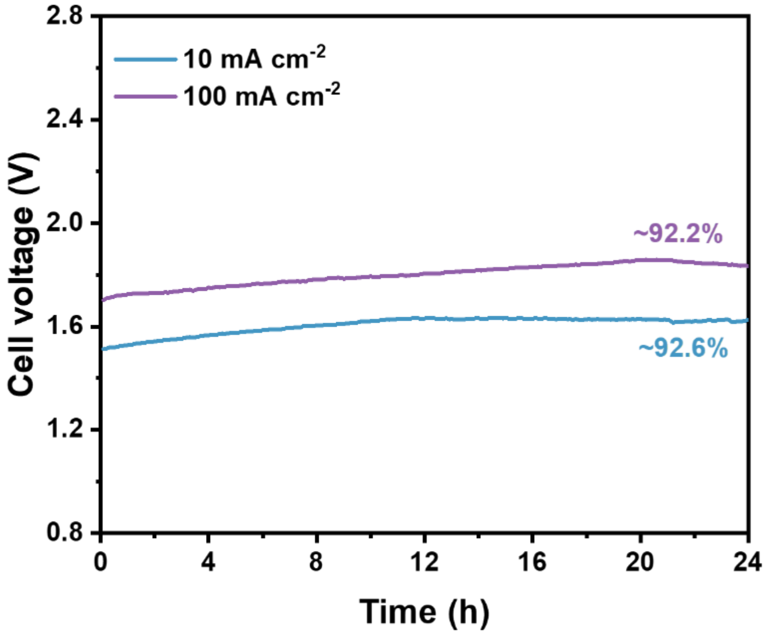


**Supplementary Figure 27**. Chronopotentiometry durability tests conducted at a fixed current density of 10 mA $cm^{-2}$ and 100 mA $cm^{-2}$ in 1.0 m KOH.

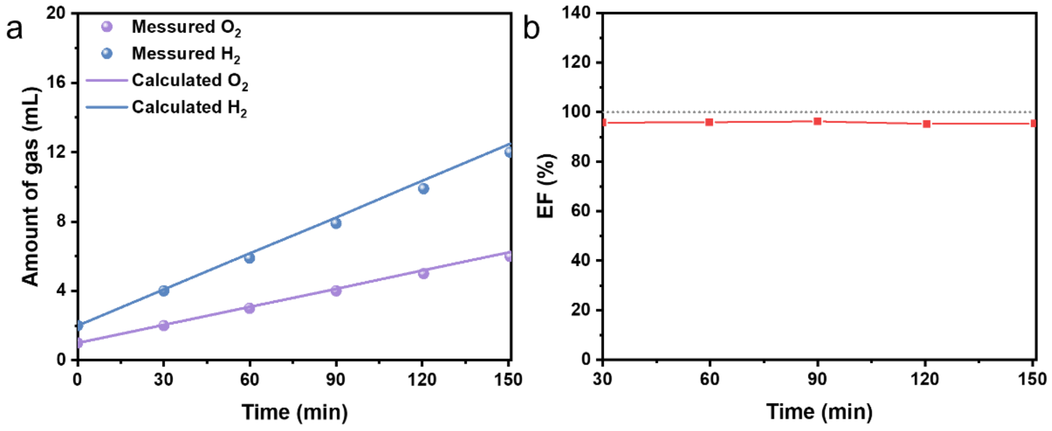


**Supplementary Figure 28**. a) Amount of gas collected and calculated for Pt/C || CoO@S-CoTe at 10 mA cm$^{-2}$. b) Faradaic efficieny of the OER at 10 mA cm$^{-2}$.

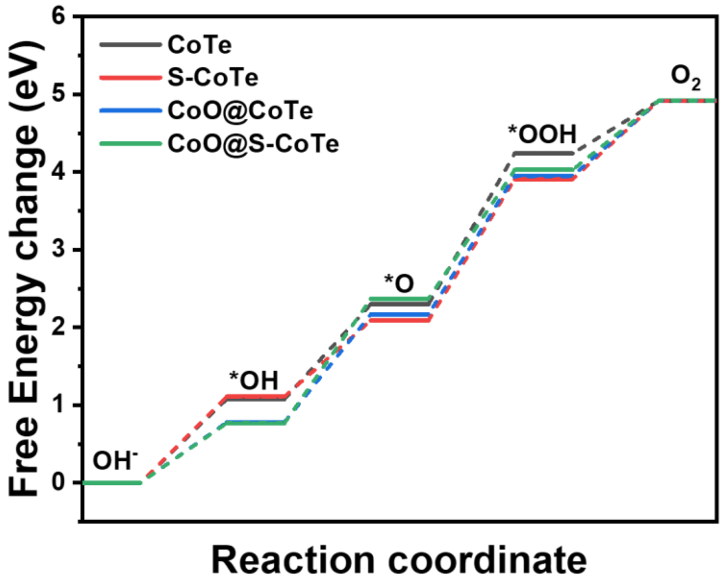


**Supplementary Figure 29**. Free energy diagrams at 0 V for the OER process on CoTe, S-CoTe, CoO@CoTe and CoO@S-CoTe.

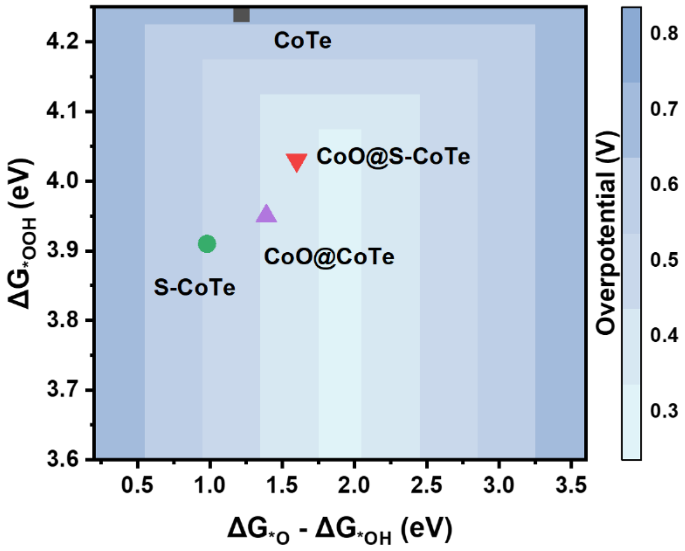


**Supplementary Figure 30**. Calculated contour volcano plot of the OER overpotential.

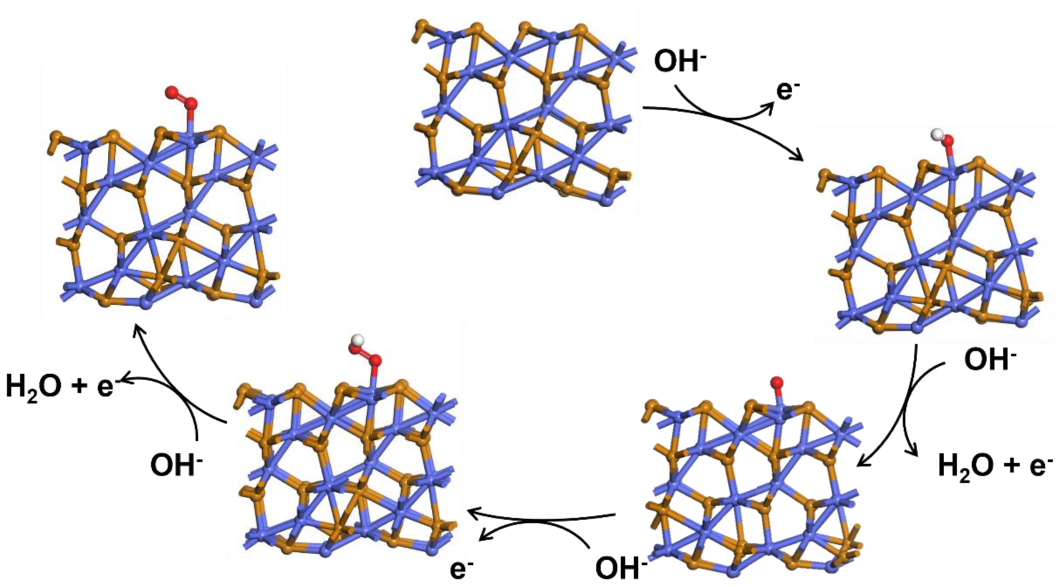


**Supplementary Figure 31**. Proposed OER mechanism of CoTe.

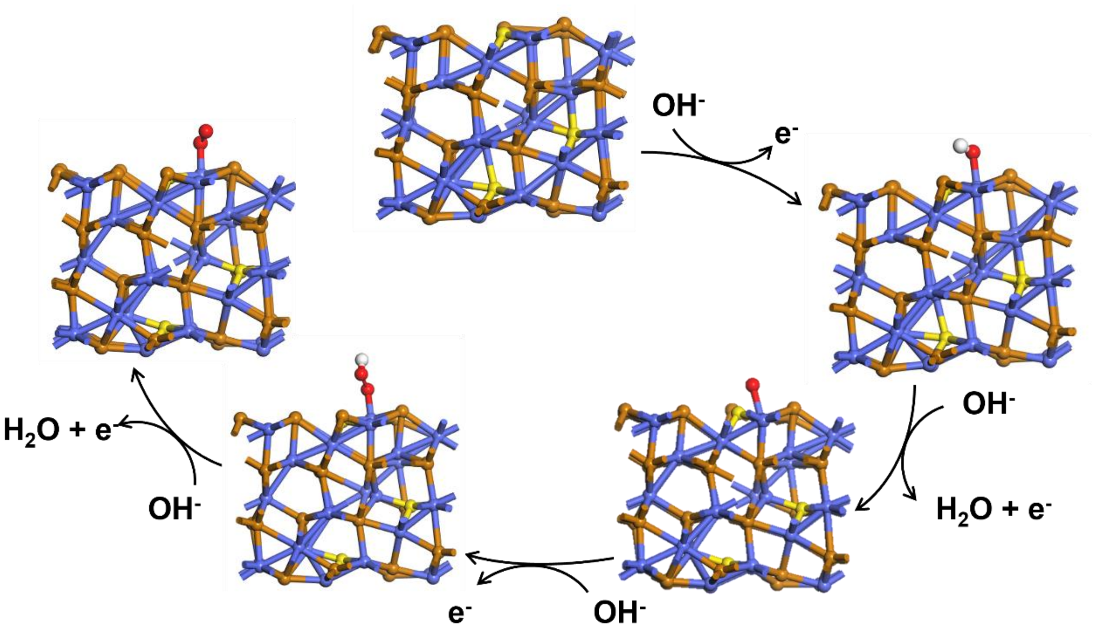


**Supplementary Figure 32**. Proposed OER mechanism of S-CoTe.

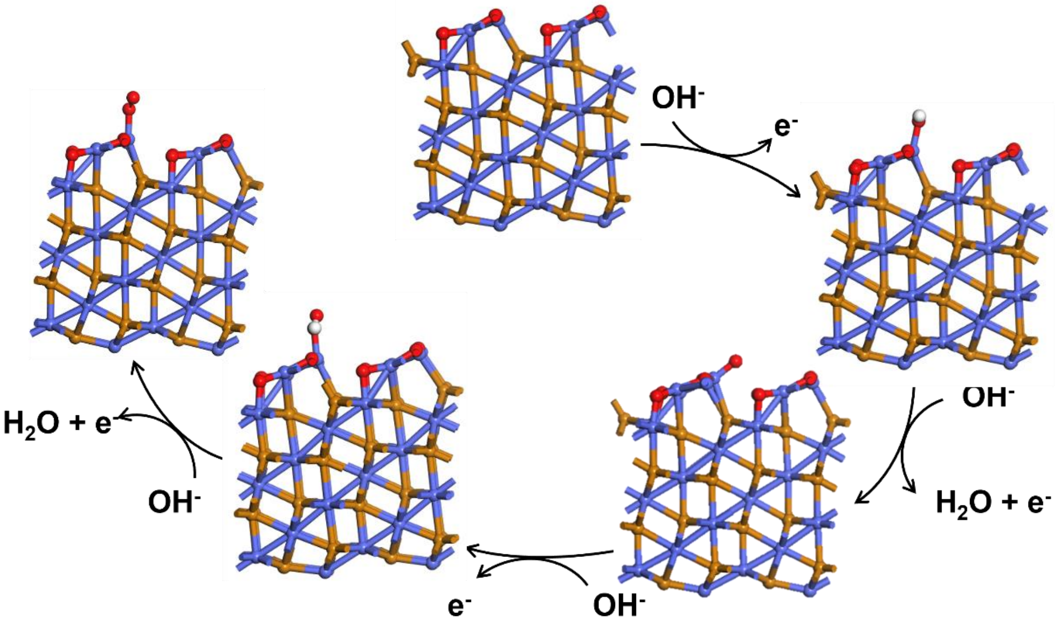


**Supplementary Figure 33**. Proposed OER mechanism of CoO@CoTe.

**Supplementary Table 1**. Element content of CoO@S-CoTe before and after stability test based on XPS data, with estimated CoO weight percent also provided.

| Element | CoO@S-CoTe | | CoO@S-CoTe after stability test | |
|---|---|---|---|---|
| | Weight (%) | Atomic (%) | Weight (%) | Atomic (%) |
| Te | 66.68 | 40.30 | 63.6 | 36.14 |
| Co | 26.81 | 35.09 | 28.96 | 35.64 |
| S | 2.81 | 6.75 | 2.42 | 5.46 |
| O | 3.7 | 17.86 | 5.02 | 22.75 |
| Total | 100.00 | 100.00 | 100.00 | 100.00 |
| $O_{lattice}/O_{total}$ (%) | 17.81 | | 8.42 | |
| CoO (wt%) | 3.08 | | 1.98 | |

**Supplementary Table 2**. Comparison of OER performance for reported electrocatalysts.

| Catalysts | Overpotential (mV) at 10 mA $cm^{-2}$ | Tafel η (mV $dec^{-1}$) | Reference |
|---|---|---|---|
| NiCo/$NiCo_2S_4$@CNS-800 | 370 | 85 | [4] |
| Fe doped CoTe | 300 | 45 | [5] |
| CoP hexagram | 269 | 61.3 | [6] |
| P-$NiSe_2$@N-CNTs/NC | 306 | 61 | [7] |
| $NiTe_2$/$Ni(OH)_2$ nanosheets | 267 | 75 | [8] |
| S,N-Co@CNT | 276 | 87 | [9] |
| Fe doped Mo/Te nanorods | 300 | 87.2 | [10] |
| $Cu_7Te_4$ nanoplates | 460 | 103 | [11] |
| Se/Fe-$Co_9S_8$-0.14 | 298 | 51.29 | [12] |
| $CoSeO_3 \cdot 2H_2O$/CC | 387 | 65 | [13] |
| o-$CoTe_2$\|P@HPC/CNTs | 241 | 46 | [14] |
| $Co(Te_{0.33}Se_{0.67})_2$ | 272 | 44 | [15] |
| $CoNiO_2$/SNC | 280 | 34 | [16] |
| $MoS_2$/CoB-Se/CC | 270 | 86 | [17] |
| ED-$FeTe_x$ | 264.4 | 54.2 | [18] |
| α-MnS | 292 | 70 | [19] |
| $CoSe_2$ nanomesh | 284 | 46.3 | [20] |
| Ir-Te nanowires | 248 | 38.1 | [21] |
| NiNCs-1T-Mn-$VTe_2$ | 258 | 48.8 | [22] |
| $RuTe_2$ | 275 | 53 | [23] |
| This Work | 246 | 56 | |

**Supplementary Table 3**. Comparison of OER performance for reported electrocatalysts with NF as substrate.

| Catalysts | Overpotential (mV) at 10 mA cm$^{-2}$ | Tafel η (mV dec$^{-1}$) | Reference |
|---|---|---|---|
| CoFe LDH/NF | 256 | 136 | [24] |
| Ni(OH)S/NF | 250 | 52.38 | [25] |
| $Fe_2OF_4$/NF | 238 | 48 | [26] |
| CoFe-$NA_2$/NF | 250 | 69.9 | [27] |
| NiYCe-MOF/NF | 245 | 65 | [28] |
| NCMO@Ag/NF | 267 | 89 | [29] |
| La–Ir NF | 263 | / | [30] |
| H-CoSx@NiFe LDH/NF | 250 | 49 | [31] |
| $Ni_3S_2$@NGCLs/NF | 271 | 99 | [32] |
| $Co_4Mo_8$/NF | 302.8 | 80.7 | [33] |
| hNC-10s/NF | 305 | 72 | [34] |
| $Fe_2B$ NPs/NF | 258 | 44 | [35] |
| NiSP/NF | 259 | 99 | [36] |
| This Work | 246 | 56 | |

**Supplementary Table 4**. Calculated Gibbs free energy values on CoTe, S-CoTe, CoO@CoTe and CoO@S-CoTe at 0V.

| Catalyst | $H_2O$→*OH | *OH→*O | *O→*OOH | *OOH→$O_2$ |
|---|---|---|---|---|
| CoTe | 1.08 | 1.22 | 1.94 | 0.68 |
| S-CoTe | 1.11 | 0.98 | 1.82 | 1.01 |
| CoO@CoTe | 0.78 | 1.39 | 1.78 | 0.97 |
| CoO@S-CoTe | 0.77 | 1.6 | 1.66 | 0.89 |

## Supplementary References


[1] a) G. Kresse, J. Furthmüller, *Physical Review B* **1996**, 54, 11169; b) G. Kresse, J. Furthmüller, *Comput. Mater. Sci* **1996**, 6, 15.

[2] a) J. P. Perdew, K. Burke, M. Ernzerhof, *Phys. Rev. Lett.* **1996**, 77, 3865; b) J. P. Perdew, M. Ernzerhof, K. Burke, *The Journal of Chemical Physics* **1996**, 105, 9982.

[3] S. Grimme, *J. Comput. Chem.* **2006**, 27, 1787.

[4] H. Yu, D. Zhang, Z. Fang, S. Xu, Q. Liu, H. Hou, L. Wang, Z. Zhou, G. Shao, W. Yang, J. Teng, S. Chen, *Electrochim. Acta* **2021**, 380, 138261.

[5] L. Zhong, Y. Bao, L. Feng, *Electrochim. Acta* **2019**, 321, 134656.

[6] Z. Liang, C. Yang, W. Zhang, H. Zheng, R. Cao, *Chin. Chem. Lett.* **2021**, 32, 3241.

[7] J. Yu, W.-J. Li, G. Kao, C.-Y. Xu, R. Chen, Q. Liu, J. Liu, H. Zhang, J. Wang, *J. Energy Chem.* **2021**, 60, 111.

[8] B. Xu, X. Yang, X. Liu, W. Song, Y. Sun, Q. Liu, H. Yang, C. Li, *J. Power Sources* **2020**, 449, 227585.

[9] P. Rao, Y. Liu, Y.-Q. Su, M. Zhong, K. Zhang, J. Luo, J. Li, C. Jia, Y. Shen, C. Shen, X. Tian, *Chem. Eng. J.* **2021**, 422, 130135.

[10] R. He, M. Li, W. Qiao, L. Feng, *Chem. Eng. J.* **2021**, 423, 130168.

[11] Q. Qin, G. Zhang, Z. Chai, J. Zhang, Y. Cui, T. Li, W. Zheng, *Nano Energy.* **2017**, 41, 780.

[12] D. Zhang, J. Zhang, X. Fu, J. Pan, Y. Wang, J. Li, B. Jiang, R. Liu, X. Wang, X. Zhang, R. Zhang, Z.-a. Qiao, *Nanotechnology* **2020**, 31, 334001.

[13] S. Anantharaj, H. Sugime, B. Chen, N. Akagi, S. Noda, *The Journal of Physical Chemistry C* **2020**, 124, 9673.

[14] Z. Chen, M. Chen, X. Yan, H. Jia, B. Fei, Y. Ha, H. Qing, H. Yang, M. Liu, R. Wu, *ACS Nano.* **2020**, 14, 6968.

[15] Y. Wang, L. Liu, Y. Wang, L. Fang, F. Wan, H. Zhang, *Nanoscale* **2019**, 11, 6108.

[16] Q. Zhang, W. Han, Z. Xu, Y. Li, L. Chen, Z. Bai, L. Yang, X. Wang, *RSC Adv.* **2020**, 10, 27788.

[17] M. Song, Y. Zhao, Z. Wu, X. Liu, *Sustainable Energy Fuels* **2020**, 4, 5036.

[18] X. Wu, L. Lu, H. Liu, L. Feng, W. Li, L. Sun, **2021**, 6, 6154.

[19] R. B. Pujari, G. S. Gund, S. J. Patil, H. S. Park, D.-W. Lee, *J. Mater. Chem. A* **2020**, 8, 3901.

[20] Y. Zhang, C. Zhang, Y. Guo, D. Liu, Y. Yu, B. Zhang, *J. Mater. Chem. A* **2019**, 7, 2536.

[21] L. Li, P. Wang, Z. Cheng, Q. Shao, X. Huang, *Nano Res.* **2022**, 15, 1087.

[22] U. N. Pan, D. R. Paudel, A. Kumar Das, T. I. Singh, N. H. Kim, J. H. Lee, *Appl. Catal., B* **2022**, 301, 120780.

[23] B. Tang, X. Yang, Z. Kang, L. Feng, *Appl. Catal., B* **2020**, 278, 119281.

[24] Z. Wang, W. Liu, Y. Hu, M. Guan, L. Xu, H. Li, J. Bao, H. Li, *Appl. Catal., B* **2020**, 272, 118959.

[25] X. Jia, H. Kang, X. Yang, Y. Li, K. Cui, X. Wu, W. Qin, G. Wu, *Appl. Catal., B* **2022**, 312, 121389.

[26] G. Zhang, Z. Li, J. Zeng, L. Yu, C. Zuo, P. Wen, Y. Liu, L. Zhong, H. Chen, Y. Qiu, *Appl. Catal., B* **2022**, 319, 121921.

[27] M. Chen, D. Liu, B. Zi, Y. Chen, D. Liu, X. Du, F. Li, P. Zhou, Y. Ke, J. Li, K. H. Lo, C. T. Kwok, W. F. Ip, S. Chen, S. Wang, Q. Liu, H. Pan, *J. Energy Chem.* **2022**, 65, 405.

[28] F. Li, M. Jiang, C. Lai, H. Xu, K. Zhang, Z. Jin, *Nano Lett.* **2022**, 22, 7238.

[29] D. Li, Y. Qin, J. Liu, H. Zhao, Z. Sun, G. Chen, D.-Y. Wu, Y. Su, S. Ding, C. Xiao, *Adv. Funct. Mater.* **2022**, 32, 2107056.

[30] W. Sun, C. Ma, X. Tian, J. Liao, J. Yang, C. Ge, W. Huang, *J. Mater. Chem. A* **2020**, 8, 12518.

[31] Y. J. Lee, S.-K. Park, *Small.* **2022**, 18, 2200586.

[32] B. Li, Z. Li, Q. Pang, J. Z. Zhang, *Chem. Eng. J.* **2020**, 401, 126045.

[33] M. Zhang, M. Chen, Y. Bi, L. Huang, K. Zhou, Z. Zheng, *J. Mater. Chem. A* **2019**, 7, 12893.

[34] Q. Liu, H. Zhao, M. Jiang, Q. Kang, W. Zhou, P. Wang, F. Zhou, *J. Mater. Chem. A* **2020**, 8, 13638.

[35] W. Wang, Y. Jiang, Y. Hu, Y. Liu, J. Li, S. Chen, *ACS Appl. Mater. Interfaces* **2020**, 12, 11600.

[36] R. A. Marquez-Montes, K. Kawashima, Y. J. Son, J. A. Weeks, H. H. Sun, H. Celio, V. H. Ramos-Sánchez, C. B. Mullins, *J. Mater. Chem. A* **2021**, 9, 7736.
